# Star-related lipid transfer protein 10 (STARD10): a novel key player in alcohol-induced breast cancer progression

**DOI:** 10.1186/s13046-018-1013-y

**Published:** 2019-01-05

**Authors:** Andrea Floris, Jia Luo, Jacqueline Frank, Jennifer Zhou, Sandro Orrù, Michela Biancolella, Sabina Pucci, Augusto Orlandi, Paolo Campagna, Antonella Balzano, Komal Ramani, Maria Lauda Tomasi

**Affiliations:** 1 Department of Medicine, Cedars-Sinai Medical Center, DAVIS Research Building 3096A, 8700 Beverly Blv, Los Angeles, CA 90048 USA; 2 Medical Genetics, Department of Medical Sciences and Public Health, University of Cagliari, Cagliari, Italy; 3 Department of Pharmacology and Nutritional Sciences, University of Kentucky College of Medicine, Lexington, KY USA; 4 Department of Biology, University of Tor Vergata, Rome, Italy; 5 Department of Biomedicine, University of Tor Vergata, Rome, Italy; 6 Casa di Cura Polispecialistica Sant’Elena, Quartu, Italy

**Keywords:** Breast cancer, Alcohol abuse, STARD10, ERBB2

## Abstract

**Background:**

Ethanol abuse promotes breast cancer development, metastasis and recurrence stimulating mammary tumorigenesis by mechanisms that remain unclear. Normally, 35% of breast cancer is Erb-B2 Receptor Tyrosine Kinase 2 (ERBB2)-positive that predisposes to poor prognosis and relapse, while ethanol drinking leads to invasion of their ERBB2 positive cells triggering the phosphorylation status of mitogen-activated protein kinase. StAR-related lipid transfer protein 10 (STARD10) is a lipid transporter of phosphatidylcholine (PC) and phosphatidylethanolamine (PE); changes on membrane composition of PC and PE occur before the morphological tumorigenic events. Interestingly, STARD10 has been described to be highly expressed in 35–40% of ERBB2-positive breast cancers. In this study, we demonstrate that ethanol administration promotes STARD10 and ERBB2 expression that is significantly associated with increased cell malignancy and aggressiveness.

**Material and methods:**

We investigated the effect of ethanol on STARD10-ERBB2 cross-talk in breast cancer cells, MMTV-neu transgenic mice and in clinical ERBB2-positive breast cancer specimens with Western Blotting and Real-time PCR. We also examined the effects of their knockdown and overexpression on transient transfected breast cancer cells using promoter activity, MTT, cell migration, calcium and membrane fluidity assays in vitro.

**Results:**

Ethanol administration induces STARD10 and ERBB2 expression in vitro and in vivo. ERBB2 overexpression causes an increase in STARD10 expression, while overexpression of ERBB2’s downstream targets, p65, c-MYC, c-FOS or c-JUN induces STARD10 promoter activity, correlative of enhanced ERBB2 function. Ethanol and STARD10-mediated cellular membrane fluidity and intracellular calcium concentration impact ERBB2 signaling pathway as evaluated by enhanced p65 nuclear translocation and binding to both ERBB2 and STARD10 promoters.

**Conclusion:**

Our finding proved that STARD10 and ERBB2 positively regulate each other’s expression and function. Taken together, our data demonstrate that ethanol can modulate ERBB2’s function in breast cancer via a novel interplay with STARD10.

**Electronic supplementary material:**

The online version of this article (10.1186/s13046-018-1013-y) contains supplementary material, which is available to authorized users.

## Background

Breast cancer is the most common invasive cancer in females worldwide**.** It accounts for 16% of all female cancers, 22.9% of invasive cancers in women and 18.2% of all cancer deaths worldwide [1]. The predictive biomarkers in breast cancer are the estrogen (ER), progesterone (PR) receptors and human epidermal growth factor receptor *HER2* (*erbB2/neu*) [2] whose overexpression is associated with a lower probability of response to tamoxifen and trastuzumab [3]*.* Currently, the endogenous and environmental factors that contribute to breast cancer etiology remain elusive, where tobacco use, unregulated diet and alcohol consumption are the three-major human cancer risk factors [4]*.* Epidemiological evidence and experimental studies support a positive association between alcohol consumption and breast cancer risk in a concentration- and duration-dependent manner, showing that alcohol drinking increases breast cancer risk by 10–20% for each glass of wine and or beer (10 g of alcohol) consumed daily by adult women [5, 6]*.* Research consistently shows that ethanol is a tumor promoter and stimulates migration/invasion as well as proliferation of breast tumor cells and enhances epithelial-mesenchymal transition [7]*,* also enhances the cell growth of existing breast tumor and its capability to invade and metastasize [8]*.* Oxidation of ethanol to acetaldehyde or formation of free radicals could be involved in ethanol-mediated breast cancer promotion, through inhibition of carcinogen-induced DNA damage repair [9, 10]*.* Cytochrome P450 2E1 (CYP2E1) is the principal P-450 responsible for the metabolism of ethanol and it has been shown to contribute to reactive oxygen species (ROS) generation in breast cancer cells [11]. However, the molecular mechanism underlying ethanol action remain to be determined. The ErbB protein family is a receptors kinase group that includes four closely related members: epidermal growth factor receptor (EGFR/ERBB1), ERBB2/neu, ERBB3 and ERBB4. ERBB2 plays a critical role in the pathogenesis of breast cancer and results amplified and/or overexpressed in 20–30% of human breast cancers correlating with poor prognosis [12]. In human breast cancer and mammary epithelial cells with high expression of ERBB2, ethanol induces ERBB2 expression and its autophosphorylation that activates the mitogen-activated protein kinases (MAPKs) signaling members, extracellular signal-regulated kinase (ERK), c-Jun NH_2_ terminal protein kinase (JNK1/2), p38 mitogen-activated protein kinase (p38 MAPK), PI3-kinase (Phosphatidyl inositol 3 kinase) and Akt (AK strain transforming), well-known to be downstream targets of ERBB2 [13]. The steroidogenic acute regulatory protein (StAR)-related lipid transfer (STARD) domain is a protein module of 210 residues that binds lipids [14]*.* STARD10 is a member of the StarD protein family and lipid transfer protein with selective binding site to phosphatidylcholine (PC) and phosphatidylethanolamine (PE), two potential precursors for lipid metabolism and a major constituent of cell membranes (REF). STARD10 is highly expressed in liver where it delivers phospholipids in the canalicular membrane for secretion into bile [15]. However, in the mammary gland, STARD10 expression is developmentally regulated for the lipids needed in milk enrichment [16]*.* Cellular growth and apoptosis may also be influenced by the PC to PE ratio as a reduction in this ratio can result in a loss of membrane integrity that could predispose to cellular transformation. Since PC is involved in membrane trafficking processes and cellular signaling, it can induce direct activation of the MEK-ERK 1/2 pathway protein, increase cell viability and induce proliferation [17]*.* The biological effects correlated with PC concentration changes in biological membranes are due to an altered cellular localization of membrane enzymatic proteins and its activation status [18]*.* The role of STARD10 as key player in subcellular lipid transfer and cellular signaling regulation has not been clarified yetPhosphorylation is a common modification that regulates the activity of proteins, increasing their local negative charge to promote conformational changes or influencing interaction with protein partners. STARD10 protein is well-known to be negatively regulated by phosphorylation via Casein Kinase II (CKII) at Serine 284 [19]*.* STARD10 is highly expressed at protein level in mouse mammary tumor, in 35% of primary breast carcinoma and in 64% of human breast cancer cell lines. This data supports the role of STARD10 as lipid binding protein in deregulated cell growth and tumorigenesis. Intriguingly, STARD10 was found to be co-expressed with ERBB2 in several breast carcinoma cell lines, suggesting a selective growth advantage and cellular transformation for tumor expressing both proteins [16]*.* Although STARD10 expression alone was not sufficient to transform cells, it potentiated cellular transformation when co-expressed with ERBB1, another member of ERBB family, by an unknown mechanism [16, 19, 20]*.* The aim of this study was to investigate the role of STARD10 and ERBB2 cross-talk in breast cancer as consequence of ethanol administration and elucidate the molecular mechanisms.

## Materials and methods

### Cell culture and treatments

All cell lines were purchased and authenticated after 30 passages from American Type Culture Collection and authentication service (ATCC, Rockville, MD), respectively. Specifically, both human breast cancer cell lines, MCF-7 (ERBB2 negative) and SKBR-3 cells (ERBB2 positive), were grown according to instructions provided by ATCC, while MCF12-A (human breast epithelial cells) were maintained in DMEM/F12 medium (Corning) containing epidermal growth factor (EGF) (20 ng/mL) (Thermo Fisher, Waltham, MA), hydrocortisone (0.5 mg/mL), cholera toxin (100 ng/mL), insulin (10 μg/mL) (Sigma, Saint Louis, MO) and supplemented with 5% horse serum (Thermo Fisher, Waltham, MA), penicillin (100 U/ml)/streptomycin (100 U/ml) at 37 °C with 5% CO2. In this study, cells were exposed to ethanol (Sigma Aldrich, St. Louis, MO) at pharmacologically relevant concentration of 100 mM for 48 h [21].

### Human breast tissue specimens

Five normal breast tissues and thirteen breast cancer tissues from surgical reductive mastoplasty and surgical resection for primary breast cancer, respectively, were used (Additional file 1: Table S1). All tissues were immediately frozen in liquid nitrogen for subsequent RNA and protein extraction. Written informed consent was obtained from each patient. The study protocol conformed to the ethical guidelines of the 1975 Declaration of Helsinki as reflected in a prior approval by Cedars Sinai Medical Center’s human research review committee.

### MMTV-neu transgenic mice model

Mice mammary adenocarcinoma tissues was provided by Dr. Jia Lou (University of Kentucky College of Medicine, Lexington, KT). FVB MMTV-neu transgenic mice were purchased from Jackson Laboratory (Bar Harbor, MA). Twelve weeks old mice were divided into two groups, the (treat group) were fed with ethanol liquid diet at concentration 6.6% *v*/v, while the other (control group) were put on an alcohol-free liquid diet. Both groups were monitored weekly to observe growth and development of tumor. The mice with the tumor that increased its size and go beyond 20 mm were euthanized and the tumor mass was analyzed [22].

### Transient cell transfection

MCF-7 and SKBR-3 cells were transfected with the following overexpression vectors: *StarD10* (Myc-DDK-tagged), *ErbB2*-EGFP, pCMV4-p65, CMV6-*c-Myc-DDK*, pMIEG3-*c-Jun*, pLX304-*Fos*-V5. All plasmids and the corresponding negative control empty vectors were purchased from Origene (Rockville, MD) and Addgene (Cambridge, MA). MCF-7 and SKBR-3 cells were cultured in 6-well plates (0.5 × 10^6^ cells/well) and transfected using 5 μl of JetPRIME from Polyplus (New York, NY) with 2 μg of target plasmid per well. After 4 h, the transfection medium was changed with regular culture medium to avoid toxicity and the cells were cultured for additional 44 h (total 48 h of transfection). Ethanol (100 mM) was administrated every 4 h to compensate its evaporation rate without replacing the culture media and mRNA and protein expression analysis were performed as indicated.

### *STARD10 and ERBB2* promoter reporter assays

The *STARD10* and *ERBB2* promoter-luciferase reporter plasmids (GeneCopoeia, Rockville, MD), *p65*, *c-Jun*, *c-Fos* and *c-Myc* were co-transfected as indicated into MCF-7 and SKBR-3 cells (0.5 × 10^6^ cells/well, 6-well plates) as described above for 24 h and ethanol (100 mM) was added as indicated for 48 h. Gaussia luciferase (GLuc) and secreted Alkaline Phosphatase (SEAP) activities were measured following the manufacturing’s instruction (GeneCopoeia, Rockville, MD).

### ChIP assay

ChIP assays were performed using Imprint Chromatin Immunoprecipitation kit (Sigma, St. Louis, MO). Sonicated chromatin was immunoprecipitated with 2 μg of antibody against p65 (Proteintech, Rosemont, IL) reverse cross-linked and PCR amplified for 35 cycles with the following murine *STARD10* promoter primer sequences: part 1. chr11:72791657–72,796,391) Forward: 5’-TCCTAATATCCAGAGGAGCAC-3′; Reverse: 5′- TCTGGAAGTTAACTGACAGCC-3′; part 2. (chr11:72791657–72,792,196) Forward: 5’-GGCTCTCAGTTAACTTCCAGA-3′; Reverse: 5’-GCACAACTAACTCAGCAGCAA-3′, and murine *ERBB2* promoter primer sequences: part 1. (chr11:98411386–98,411,757) Forward: 5’-GAAAGTAGATTAAGAGAGGGCC-3′; Reverse: 5’-GTTCTGACTTTACCCAGTTCTC-3′ (Ambion, Austin, TX). Human STARD10 promoter primer sequences are: Forward 5’-CTTGAGCTCCTGAGAAATGTAGT-3′; Reverse 5’-GAGGGTCATTCCTTGTAATCAT-3′, while human ERBB2 promoter primer sequences are: Forward 5’-CACAAGGTAAACACAACACATCC-3′; Reverse 5’-GTAAAGGGCCCCGTGGGAA-3′.

### RNA interference

To perform the RNAi experiments, five different predesigned small interfering RNAs (siRNAs) targeting human *STARD10* (#1 sense sequence: 5’-GGCCAUGAAGAAGAUGUACtt-3′, antisense: 3’-GUACAUCUUCUUCAUGGCCtt-5′), (#2 sense sequence 5’-GGCCAUGAAGAAGAUGUACtt-3′ and antisense: 3’-GUACAUCUUCUUCAUGGCCtt-5′), (Ambion, Austin, TX),and human *RELA* (#1 sense sequence: 5’-GCCCUAUCCUUUACGUCAtt-3′, antisense: 3’-UGACGUAAAGGGAUAGGGCtg-5′), (#2 sense sequence: 5’-GGAGUACCCUGAGGCUAUAtt-3′, antisense: 3’-UAUAGCCUCAGGGUACUCCat-5′) and negative control siRNA were purchased from Ambion (Austin, TX), while two human ERBB2 siRNAs were obtained from Qiagen (Hilden, Germany) (#1 catalog no. SI02223571; #2 catalog no.SI00300195). MCF-7 and SKBR-3 cells were cultured in 6-well-plate (0.5 × 10^6^ cells/well) and transfected using RNAiMax (5 μl/well) (Invitrogen, Carlsbad, CA) with *STARD10* siRNA (10 nM), ERBB2 siRNA (10 nM), *RELA* siRNA (10 nM) or negative control siRNA for 48 h for mRNA or protein expression analysis. For combined overexpression and silencing, overexpression was performed in the last 24 h of *STARD10, RELA or ERBB2* silencing.

### Real-time PCR analysis

Total RNA was isolated using Quick-RNA Kits (Zymo Research, Irvine, CA), according to the manufacturer’s protocol, subjected to reverse transcription (RT) by M-MLV Reverse transcriptase (Invitrogen, CarlsBad, CA). Two μl of RT product was subjected to real-time PCR analysis. TaqMan probes for human *STARD10*, *ERBB2*, *RELA*, *c-Myc*, *c-Fos* and *c-Jun* and the Universal PCR Master Mix were purchased from ABI (Foster City, CA). Hypoxanthine phosphoribosyl-transferase 1 (*Hprt1*) was used as housekeeping gene. The delta Ct (ΔCt) obtained was used to find the relative expression of genes according to the formula: relative expression = 2-ΔΔCt, where ΔΔCt = ΔCt of respective genes in experimental groups – ΔCt of the same genes in control group.

### Western blots

Proteins from MCF-7, SKBR-3 cells and animal breast tissues were prepared using RIPA buffer containing protease inhibitor cocktail (Sigma, St. Louis, MO) and resolved on 10% SDS- polyacrylamide gels following standard protocols (Amersham BioSciences, Piscataway, NJ). Membrane were blotted with STARD10, ERBB2, ERK, phospho-ERK, c-MYC, p65, c-JUN, c-FOS (Proteintech, Rosemont, IL), control β-actin and Histone 3 (Sigma, St. Louis, MO) antibodies. Membranes were developed by chemiluminescence ECL detection system (Amersham BioSciences, Pittsburgh, PA) and blots were quantified using the Quantity OneTM densitometry program (Bio-Rad laboratories, Hercules, CA).

### Immobilized metal affinity

Cells were plated in 75cm^2^ Flask (Corning, NY) (~ 60–80% confluency) and treated with ethanol (100 mM) for 48 h. Thus, cells were detached from the culture plate using 0.25% Trypsin-EDTA (Fisher Scientific, Hampton, NH) and collected by centrifugation at 1000 RPM × 2 min. The total proteins were extracted as described above and subjected to immobilized metal affinity chromatography using the PhosphoCruz Protein Purification Columns (Santa Cruz Biotechology, Dallas, TX) according to the manufacturer’s protocols. The phosphoenriched lysates were subjected to immunoblotting using STARD10 monoclonal antibody.

### Cell proliferation and viability

The MTT assay was performed to determine the number of viable cells in culture using the Cell Counting Kit-8 (Bimake.com, Houston, TX). MCF-7 and SKBR-3 cells were plated into 96-well-plates (4x10^3^cells/well). 1/10 volume of MTT labeling reagent was added to each well and incubated at 37 °C for 4 h until the color turned orange. Plate reader was used to measure absorbance of formazan product at 570 nm, with a reference wavelength of 750 nm.

### Cell migration assays

Cell migration assay was performed using IBIDI Culture-Inserts (2-well) (Ibidi, Munich, Germany). MCF-7 and SKBR-3 were plated at a concentration of 5 × 10^4^ cells per 70 μL culture media, and after 24 h of incubation, culture inserts were removed. Photographs of the movement of cells into the scratch area were taken every 24 h until the scratch area had closed using EVOS XL Imaging System (Life Technologies, Carlsbad, CA). Wound healing was then analyzed using ImageJ software (https://imagej.nih.gov/ij/). Each assay was repeated in triplicate.

### Measurement of intracellular calcium

Intracellular calcium levels were determined with a colorimetric calcium detection kit from Abcam (Cambridge, MA). Briefly, cells grown on 10 mm dishes and breast tissues from animal model were lysate and centrifuged at 15,000 RPM for 15 min at 4 °C. The supernatant was collected and reacted with chromogenic reagent. The absorbance of formed chromophore was measured at 575 nm using the SPECTROstar Omega reader (BMG Labtech, Ortenberg, Germany).

### Membrane fluidity assay

The membrane fluidity kit from Marker Gene Technologies (Eugene, OR) was used to measure the relative membrane fluidity in MCF-7 and SKBR-3 cells according to the manufacturer’s protocol. Approximately 5 × 10^5^ cells were seeded into 4-well Chamber slides (Thermo Fisher, Waltham, MA), treated with ethanol (100 mM for 48 h) and transfected with *STARD10* plasmid as described above. The slides were treated with 200 μl of perfusion buffer with 20 μM fluorescent lipid reagent (pyrene decanoic acid) and 0.08% of pluronic F127. After 1-h incubation the cells were washed twice with PBS and we recorded fluorescence emissions between 392 and 450 nm in 2 nm steps after excitation at 360 nm with the FLUOstar Omega (BMG Labtech, Ortenberg, Germany). With increased membrane fluidity, the lipophilic pyrene probe forms excimers upon interaction. The ratio of excimer (peak around 450 nm) to monomer (peak around 394–398 nm) IE/IM was calculated as a quantitative measure of membrane fluidity.

### Casein kinase II activity assay

Casein Kinase II activity was measured in MCF-7, SKBR-3 breast cancer cells (1 × 10^6^ cells/well) and 10 mg of mice breast tissues lysate using the CycLex CK2 Kinase assay kit (Woburn, MA) according to the manufacturer’s recommended protocol.

### Statistical analysis

Data are expressed as mean ± SEM. Statistical analysis was performed using ANOVA and Fisher’s test. For mRNA and protein levels, ratios of genes and proteins to respective housekeeping densitometric values were compared. Significance was defined by *p* < 0.05.

## Results

### STARD10 expression in normal human breast and cancer tissues

**Fig. 1:**
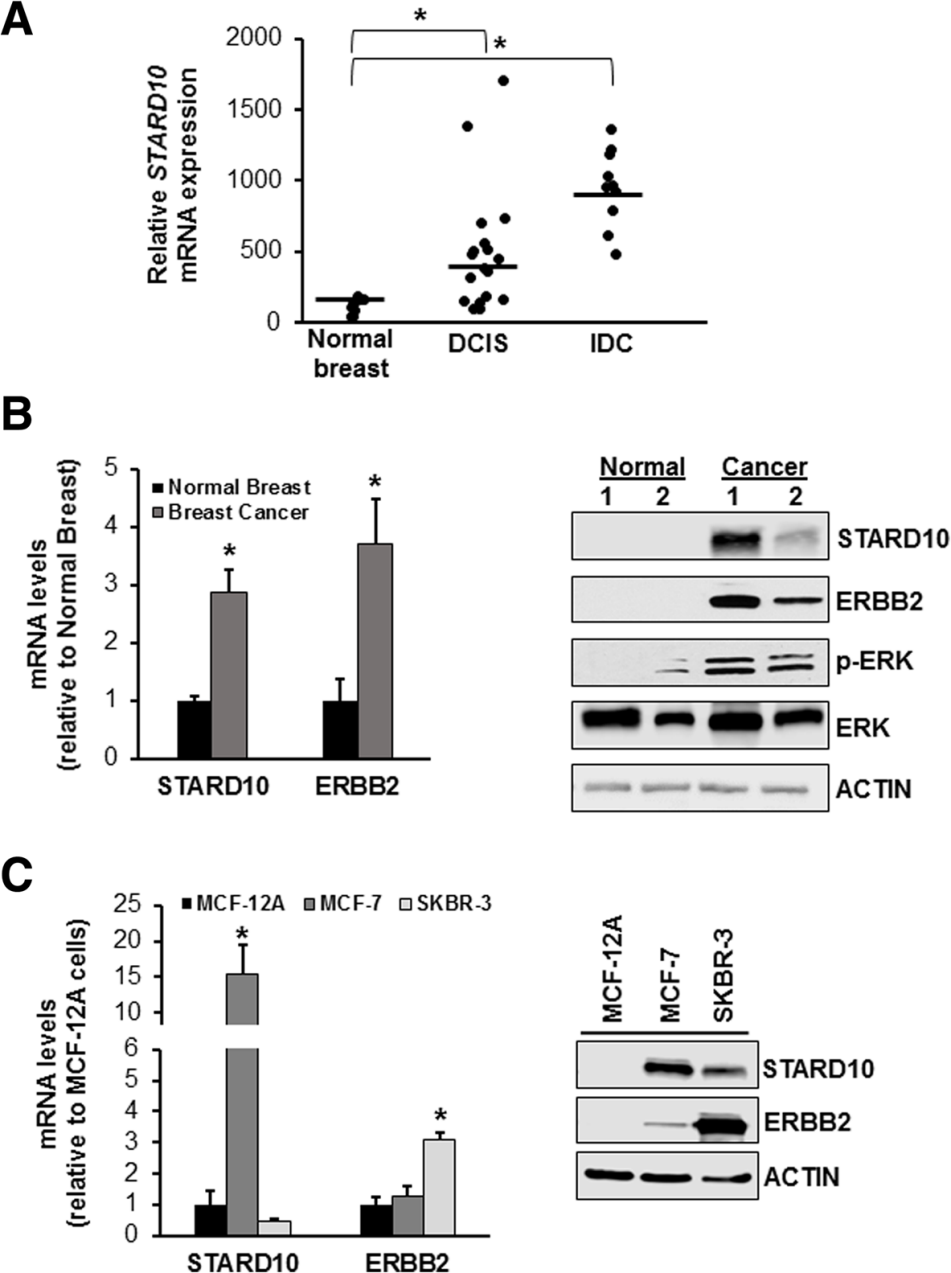
Expression of STARD10 in breast cells and tissues. **a** *STARD10* mRNA level from 28 independent breast cancer microarrays of DCIS and IDC (GEO database) compared to normal breast tissue. The database normalized STARD10 expression by RMA (robust multi-array average) method. *p < 0.001 vs. normal breast. **b** RT-PCR analysis of STARD10 and ERBB2 expression in 10 primary ERBB2-positive human breast tumors compared to normal breast tissues. Western blotting analysis of STARD10, ERBB2, pERK, and ERK (right panel). Results represent mean ± SE from 15 samples. *p < 0.04 vs. normal breast. **c**MCF-12 cells were used as negative control. mRNA levels of *STARD10* in MCF-7 and SKBR-3 cells were compared to mRNA expression in MCF-12 cells using RT-PCR. Western blotting was performed to measure STARD10 and ERBB2 protein levels. Results are expressed as a fold relative to control (mean ± SE) from 10 experiments. *p < 0.002 vs. STARD10 MCF-12 cells

Because STARD10 expression appears to be deregulated in several types of cancer including breast cancer [16], we examined STARD10 mRNA level in 38 independent breast cancer microarray datasets from the GEO database (Additional file 2: Table S2). *STARD10* mRNA levels were at least 5- and 10-fold higher in DCIS and IDC, respectively, then normal breast tissues (Fig. 1a). Consistent with these results, all 13 ERBB2-positive breast tumors (Additional file 1: Table S1) that we tested had 3- and 4-fold higher levels of *STARD10* and *ERBB2* mRNA, respectively, compared to normal breast tissue (Fig. 1b**,** left panel). In normal breast tissues, STARD10 expression was not detectable at protein level, while in ERBB2 positive human breast cancer tissue, it was expressed in 30% of samples (Fig. 1b**,** right panel). To confirm that high level of ERBB2 expression correlates with its downstream targets, we next measured ERK and p-ERK protein levels in the same human tissue samples that indicated both proteins levels increased compared to control (Fig. 1b**,** right panel). This data supports the literature which states that STARD10 is overexpressed in 35% of primary human breast cancers and positively correlates with ERBB2 overexpression [16, 20].

### STARD10 and ERBB2 expression in human breast cell lines

All cell lines that overexpressed *ERBB2* mRNA were found to have high STARD10 levels. STARD10 expression, however, was also detected in cell lines that did not overexpress ERBB2 [16]. Here, we confirmed that STARD10 was highly expressed independently of ERBB2 level (Fig. 1c). Specifically, both MCF-7 and SKBR-3 cells exhibited a gain of *STARD10* protein level even though its mRNA level appeared to be upregulated only in MCF-7 cells, compared to normal MCF-12A cells (Fig. 1c). This finding confirmed the immunohistochemical analysis that show STARD10 expression was negligible in normal breast tissue [16]. Alterations in hormone homeostasis during breast cancer transformation may be responsible for the induction in STARD10 expression even though no evidence is presented so far.

### Alcohol administration enhances STARD10 protein level in MMTV-neu transgenic mice and in breast cancer cell lines

**Fig. 2:**
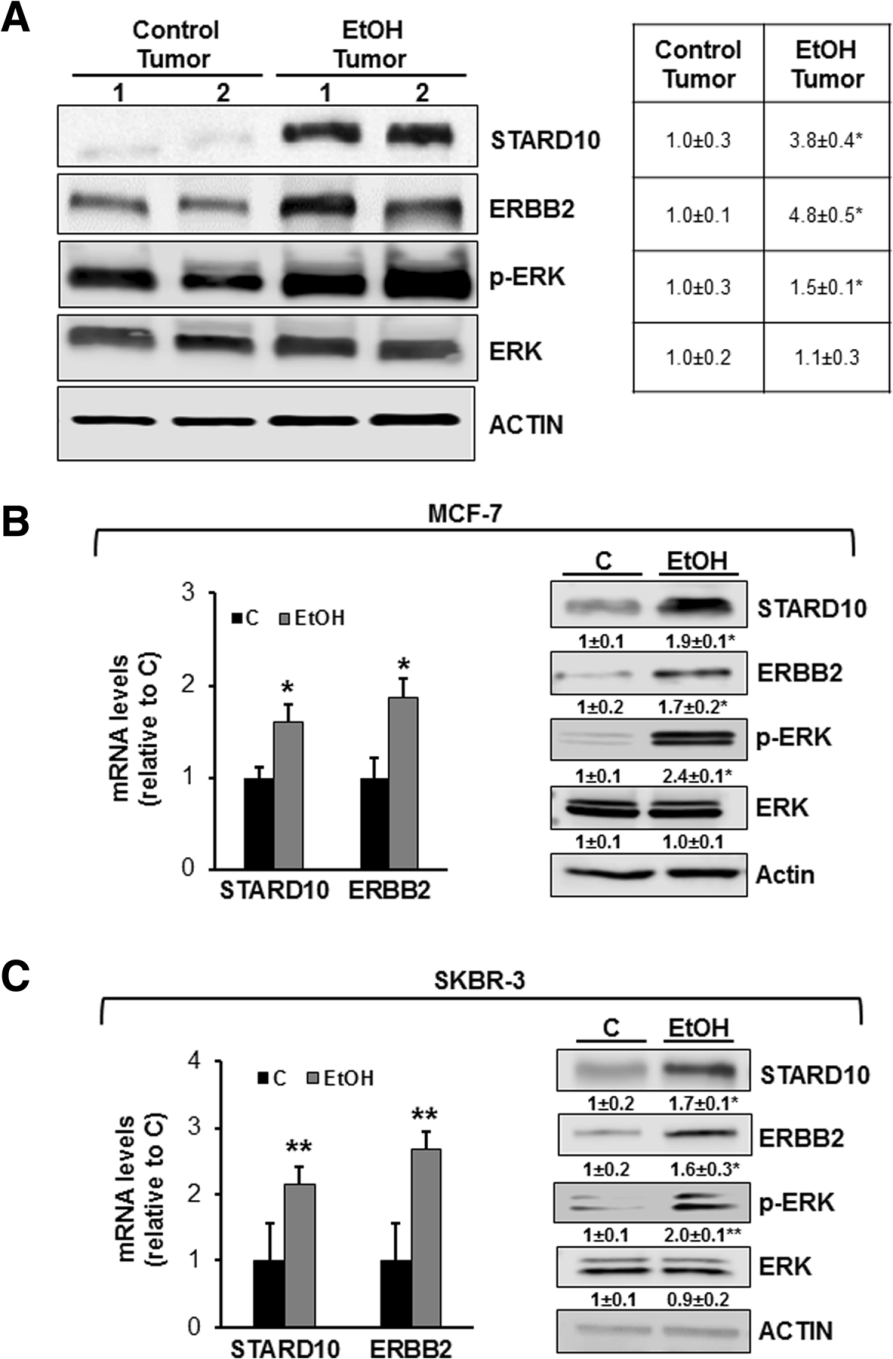
Ethanol induces STARD10 expression in vivo and in vitro. **a** Western blotting analysis of STARD10 and ERBB2 expression in ethanol-fed MMTV-neu transgenic mice. Densitometric ratios normalized to actin are shown in the right panel. Results are expressed as fold relative to control (mean ± SE) from 4 mice per group. *p < 0.05 vs. control group. **b, c** MCF-7 and SKBR-3 cells were plated at density of 0.4 × 10^6^ cells in 6-well/plates and treated with 100 mM ethanol for 48 h. The mRNA levels of *STARD10* and *ERBB2* in ethanol-treated MCF-7 and SKBR-3 cells were measured by RT-PCR and compared to control. STARD10, ERBB2, pERK, and ERK were analyzed using Western blotting. Results are expressed as fold relative to control (mean ± SE) from 5 experiments. *p < 0.02 vs control left panel; *p < 0.01 vs. control rigth panel

Luo’s laboratory demonstrated that alcohol feeding in the FVB MMTV Neu transgenic mice, that express high levels of neu (ERBB2 in human), increased cancer metastasis activating ErbB2/p38γ MAPK signaling pathway [22]. Here we investigated whether alcohol influences STARD10 expression in the above animal model. Alcohol administration increased STARD10, ERBB2 and p-ERK protein levels by 6.8-, 4.8- and 1.5-fold compared to control tumor tissues (Fig. 2a). Ethanol administration in ERBB2 negative MCF-7 cell line promoted the expression of *ERBB2* itself in these cells converting them to a ERBB2 positive state that was associated with a 1.6-fold induction in STARD10 mRNA (Fig. 2b). ERBB2 positive SKBR-3 cells also responded positively to ethanol treatment by enhancing both STARD10 and ERBB2 mRNA levels (2.2 and 2.6-fold compared to controls, Fig. 2c). This correlates well with our in vivo mouse tumor data where ERBB2 positivity is observed (Fig. 2a). A comparable induction in STARD10 and ERBB2 protein levels was observed upon ethanol exposure in MCF-7 and SKBR3 cells (Fig. 2b and c). Activated ERK (p-ERK) regulates growth factor-responsive targets in the cytosol and it is well-known function downstream of ERBB2 [23]. Hence, we evaluated its activation status in vitro. p-ERK was found significantly increased by 2-fold after alcohol treatment compared to control (Fig. 2b and c) as we previously found in the in vivo model (Fig. 2a).

### STARD10-ERBB2 crosstalk upon ethanol treatment in vitro and in vivo

**Fig. 3:**
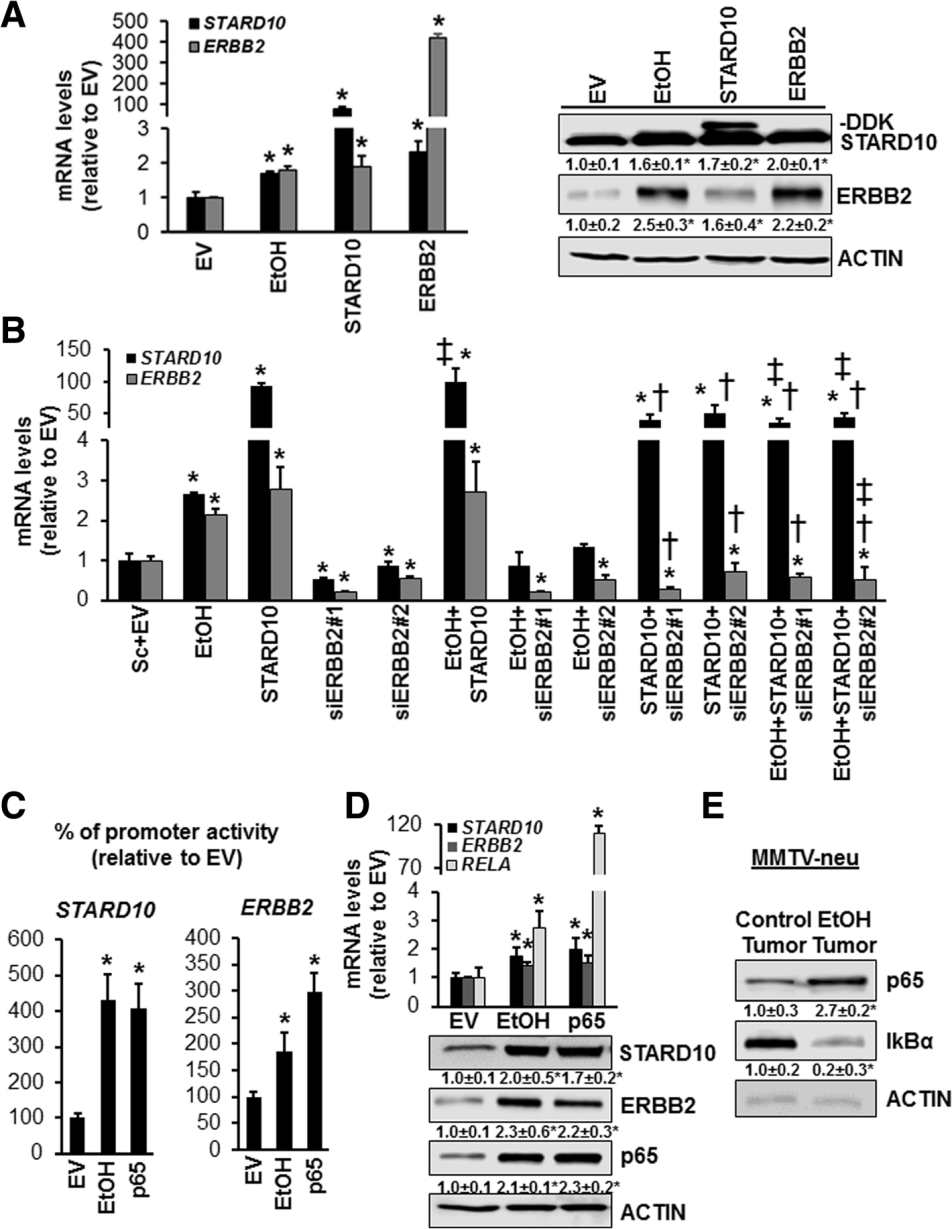
Ethanol-induced p65 positively regulates STARD10 and ERBB2 expression in MCF-7 and MMTV-neu. **a** mRNA analysis of *STARD10* and *ERBB2* was accomplished using RT-PCR in MCF-7 cells. 0.4 × 10^6^ cells were transfected and treated with 100 mM ethanol for 48 h. Western blotting analysis was performed to measure STARD10 and ERBB2 compared to control from 4 indipendent experiments. *p < 0.04 vs. EV. †*p* < 0.05 vs STARD10 or ERBB2. **b** mRNA levels of STARD10 and ERBB2 in MCF-7 cells treated with ethanol (100 mM) and transfected with STARD10 overexpression vector and ERBB2 siRNA (10 nM) for 48 h. Results are expressed as fold relative to Sc + EV (mean ± SE) from 3 independent experiments. *p < 0.04 vs.Sc + EV; †*p* < 0.04 vs. STARD10; ‡*p* < 0.03 vs. EtOH. **c** *STARD10* and *ERBB2* promoter activity analysis in MCF-7 cells using reporter assay from 4 indipendent experiments. *P < 0.04 vs. EV STARD10 promoter. **p < 0.05 vs. EV ERBB2 promoter. **d** upper panel. RT-PCR analysis of STARD10 and ERBB2 expression. Cells were treated with 100 mM ethanol or p65 transfection. *p < 0.04 vs. EV. Lower panel. Western blotting analysis of STARD10, ERBB2, p65. Results are expressed as fold relative to EV (mean ± SE) from 3 independent experiments. *p < 0.05 vs. EV. **e** Western blotting analysis of p65 and IkBα in ethanol-treated MMTV-neu mice. Results are expressed as fold relative to control (mean ± SE) from 4 mice per group. *p < 0.05 vs. control tumor

Since our preliminary data proves that ethanol treatment causes STARD10 and ERBB2 upregulation in vivo and in vitro (Fig. 2), we further explored the role of STARD10 in ethanol-induced tumor promotion to test the hypothesis that STARD10 and ERBB2 cooperate in ethanol induced breast cancer. We overexpressed *STARD10* for 24 h in vitro which caused 3- and 1.6-fold induction of *ERBB2* mRNA levels in MCF-7 and SKBR-3 cell lines, respectively, when compared with empty vector control (Fig. 3a), while forced expression of *ERBB2* caused a 2- and 1.8-fold increase in *STARD10* mRNA level in MCF-7 cells (Fig. 3a) and SKBR-3 (Additional file 3: Figure S1A), compared to control vector. Similar results were found at protein levels. Specifically, STARD10 and ERBB2 overexpression raised the level of ERBB2 protein by 1.6- and 1.8-fold in MCF-7 cells (Fig. 3a) and SKBR-3 cells (Additional file 3: Figure S1A), respectively, compared to empty vector control. Intriguingly, we found that 48 h *ERBB2* knockdown lowers the endogenous mRNA level of STARD10. Moreover, ethanol requires ERBB2 to induce STARD10 expression in both MCF-7 and SKBR-3 cell lines (Fig. 3b and Additional file 3: Figure S1B). This finding suggests that STARD10 and ERBB2 positively regulate each other’s expression in breast cancer cells.

### Ethanol-induced p65 expression promotes STARD10 and ERBB2 expression in vivo and in vitro

The stress-responsive transcription factor NF-κB is activated by a variety of cytotoxic conditions and it is considered to be the major downstream event of ERBB2 overexpression [24]. In order to investigate whether p65 was involved in ethanol-induced STARD10 and ERBB2 expression, PROMO™ software [25] was used to predict the transcriptional factors (TFs) that could potentially bind and regulate both STARD10 and ERBB2 promoters. We provided evidence that in human *STARD10* promoter, p65, c-MYC, c-FOS and c-JUN are the predominant TFs that co-occupy this region (chr11:72791657–72,795,657) (Additional file 4: Figure S2A). All the above overexpressed TFs positively regulated STARD10 expression (Additional file 4: Figure S2B and S2C) in MCF-7 cell except c-JUN even though several binding sites were found in *STARD10* promoter sequence (Additional file 4: Figure S2A). One of the more interesting finding was that p65 had the stronger induction at protein level of STARD10 compared to the other TFs (Additional file 4: Figure S2D**)**. Alcohol consumption is associated with higher expression of NF-kB p65 that stimulates tumor growth and aggressiveness [26]. Indeed, p65 overexpression had similar effect as ethanol treatment on STARD10 and *ERBB2* promoter activities that were induced by 4- and 3-fold in both MCF-7 and SKBR-3 cell lines, respectively, compared to empty vector. This was associated with a corresponding increase in *STARD10* and *ERBB2* expression levels (Fig. 3c-d-e and Additional file 3: Figure S1C-D). This finding was also confirmed analyzing the p65 protein level in the FVB MMTV Neu transgenic mice, where it was strongly induced by 2.7-fold in ethanol-fed mouse tumor compared to control tumor and the p65 nuclear translocation inhibitor, IkappaB-alpha (IkBα) [27] was reduced by 80% compared to control (Fig. 3e).

**Fig. 4:**
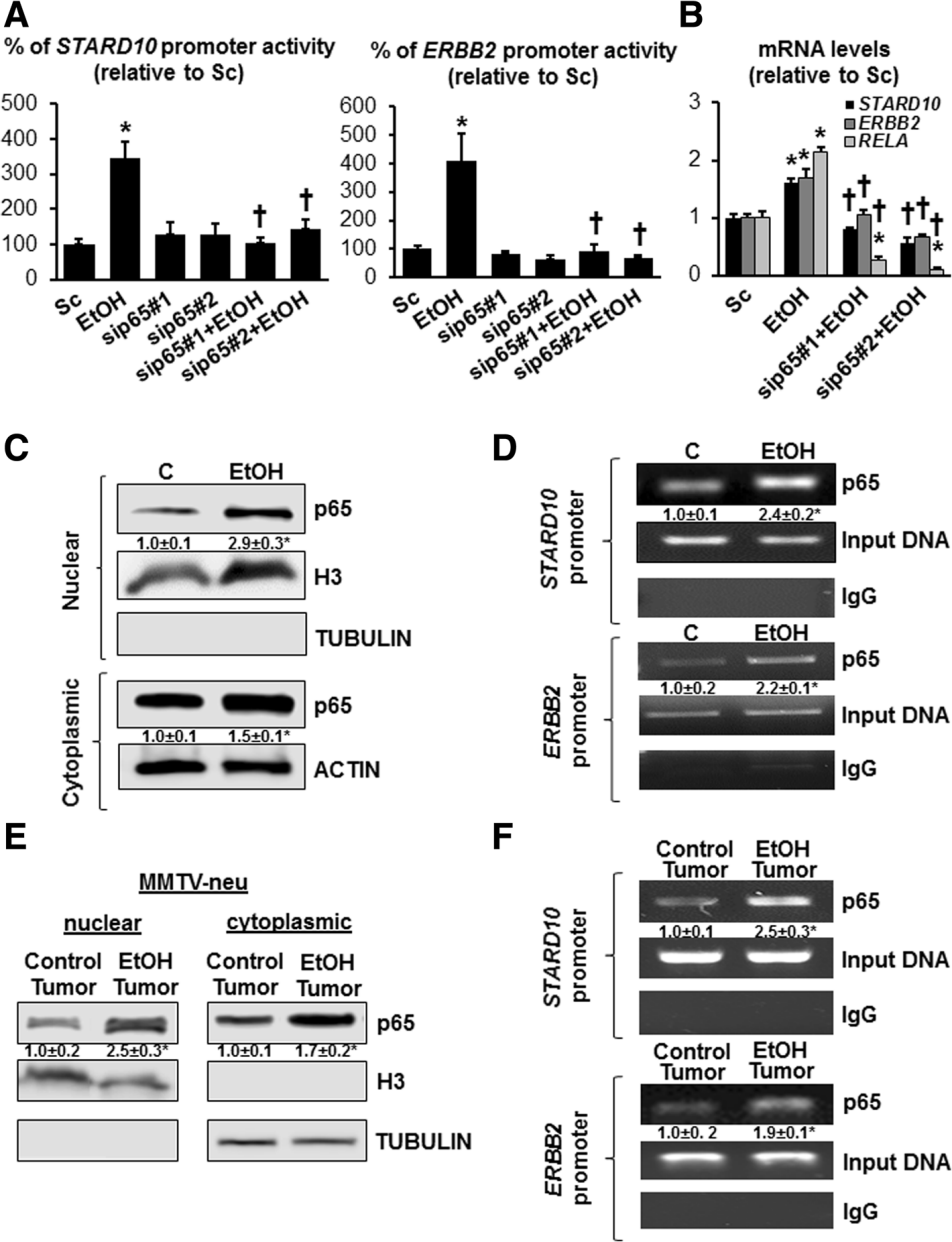
p65 positively regulates ethanol-induced STARD10 and ERBB2 expression binding their promoter sequences in MCF-7 cells. **a** *STARD10* and *ERBB2* promoter activity assay and RT-PCR for STARD10 and ERBB2 mRNA levels were measured in MCF-7 cells treated with 100 mM ethanol and transfected with sip65 (10 nM) for 48 h.. *p < 0.03 vs. Sc. †*p* < 0.04 vs. EtOH for STARD10 promoter; *p < 0.04 vs. Sc. †*p* < 0.04 vs. EtOH for ERBB2 promoter. *p < 0.004 vs. Sc; †*P* < 0.03 vs. EtOH for mRNA levels. **b** mRNA levels of *STARD10*, *ERBB2* and *RELA* measure by RT-PCR from 3 indenpendet experiments. *p < 0.004 vs. Sc; †*p* < 0.03 vs. EtOH. **c** Western blotting analysis of p65 in nuclear and cytoplasmic fractions. Nuclear marker (H3) and cytosolic marker (tubulin) were immunoblotted to demonstrate fraction purity. Data are expressed as (mean ± SE) from triplicate of 4 independent experiments. *p < 0.05 vs. control. **d** ChIP analysis for p65 binding to *STARD10* and *ERBB2* promoters. Input genomic DNA (Input DNA) was used as a positive control, while non-specific antibody IgG was used as a negative control. Results are summarized as the densitometric changes as fold of control after normalizing to input DNA. *p < 0.03 vs. control. **e** Western blotting analysis of p65 from nuclear and cytoplasmic fractions from ethanol-fed MMTV-neu transgenic mice tumor. Results are expressed as fold relative to control tumor (mean ± SE) from 4 mice per group. *p < 0.05 vs. control tumor. **f** ChIP analysis of p65 binding to ERBB2 and STARD10 promoters in MMTV-neu ethanol-fed transgenic mice tumor. Results are summarized as the densitometric changes as fold of control after normalizing to input DNA. *p < 0.04 vs. control

In order to demonstrate that ethanol positively regulates both STARD10 and ERBB2 expression via p65 involvement, we performed the *RELA* gene silencing in vitro to test our hypothesis. Figure 4a and Additional file 5: Figure S3A clearly show that ethanol required p65 to induce *STARD10* and *ERBB2* promoter activities in both MCF-7 and SKBR-3 cell lines, in addition this trend was confirmed by measuring the mRNA levels of these two genes (Fig. 4b and Additional file 5: Figure S3B). This data also confirmed previously published findings showing the ability of p65 to bind and regulate ERBB2 promoter [28].

### Ethanol promotes p65 nuclear translocation and its binding to STARD10 and ERBB2 promoter sequences

Since NF-κB is also an important redox-sensitive TF and ethanol increased intracellular ROS level [29, 30], we postulated that ethanol activates NF-κB signaling. NF-κB activation is associated with nuclear translocation of the p65 component of the complex and IκBα phosphorylation and degradation [31]. As show in Fig. 4c and in Additional file 5: Figure S3C, ethanol induced both nuclear and cytoplasmic p65 NF-κB protein levels by 2.9- and 1.5-fold in MCF-7 and by 1.6- and 1.3-fold in SKBR-3, respectively, indicating that ethanol stimulated also total p65 in addition of nuclear translocation of p65 NF-κB. Ethanol also enhanced IκB-α decreased the levels of IκB-α. This finding was also confirmed in vivo ethanol treated MMTV-neu mice (Fig. 4e). Furthermore, we demonstrated that ethanol treatment strongly induces p65 binding to both *STARD10* and *ERBB2* promoter sequences in MCF-7 cells by 2.4- and 2.2-fold, and in MMTV-neu mice by 2.5- and 1.9-fold, respectively (Fig. 4d and f). These results indicated that ethanol exposure activated NF-κB signaling on both *STARD10* and *ERBB2* promoters in breast cancer cells in vitro and in vivo.

### Ethanol lowers CKII activity in breast cancer

**Fig. 5:**
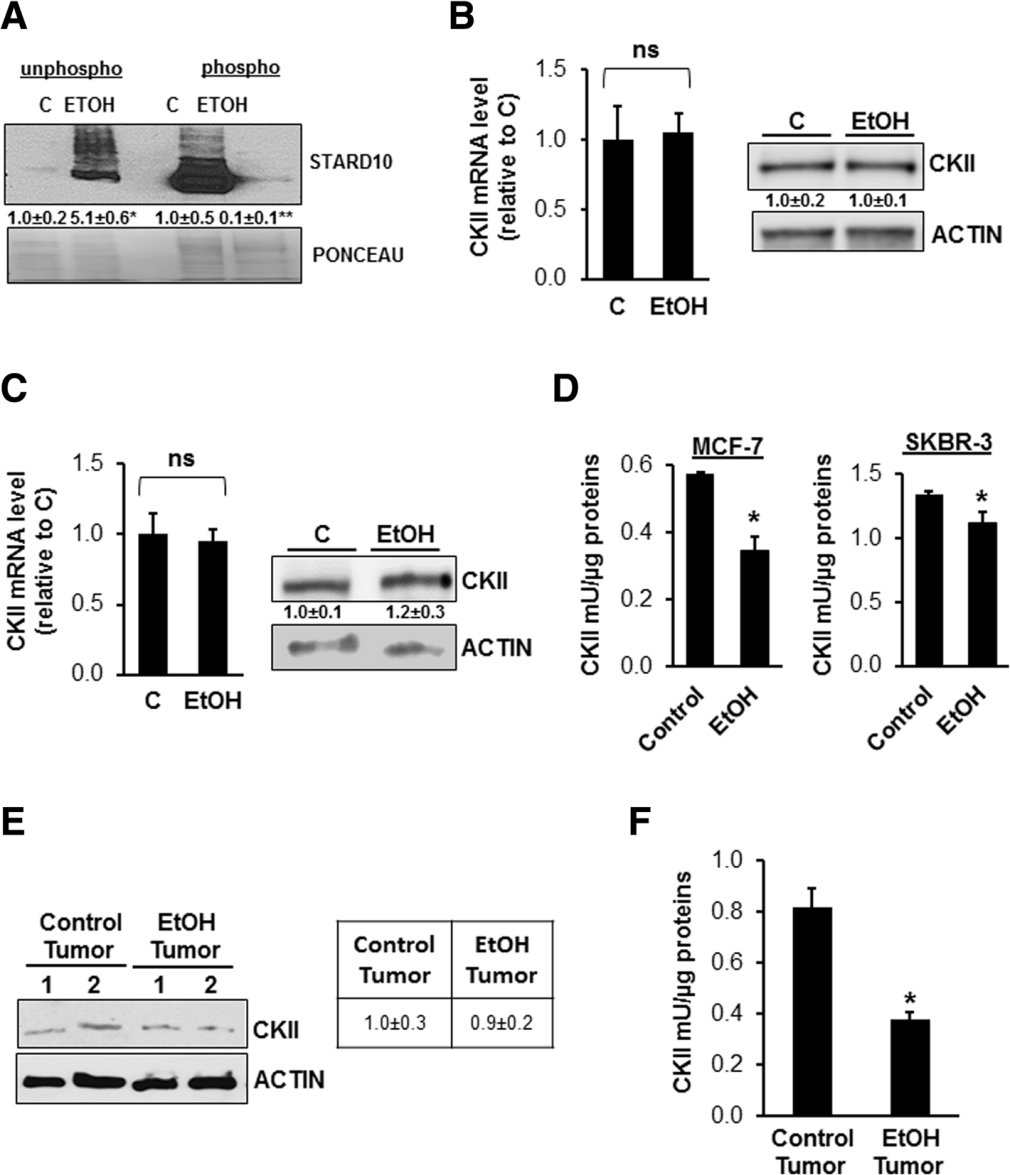
Effect of ethanol on CKII expression and/or activity in vitro and in vivo. **a** phosphorylated and unphopshorylated STARD10 in ethanol-treated MCF-7 cells protein extracts (100 mM for 48 h). STARD10 protein levels were analyzed by Western Blotting . The results are expressed as fold relative to control from 3 independent experiments and normalized with red pounceau. **b, c** CKII mRNA and protein levels were measured by RT-PCR and Western blotting, respectively in MCF-7 and SKBR-3 cell lines. CKII protein was normalized with β-actin expression. Three independent experiments were performed in triplicates. **d** The CKII enzymatic activity was evaluated as absorbance at 450 nm of cell lysate in modified kinase reaction buffer and normalized with standard in MCF-7 ans SKBR-3 cells treated with 100 mM ethanol for 48 h. Data are expressed as (mean ± SE) from 4 independent experiments performed in triplicates. *p < 0.01 vs. control. **e** Western blotting analysis of CKII in ethanol-fed MMTV-neu mice tumors. Densitometric ratios normalized to actin is shown in the right panel. Four mice per group were used. **f** CKII enzymatic activity assay in MMTV-neu was normalized with standard from 4 mice per group. *p < 0.005 vs. control tumor

CKII has been described to be a key negative regulator of STARD10 modulating is phosphorylation status [19]. In order to explore the role of ethanol on STARD10 phosphorylation/activation, MCF-7 and SKBR-3 cell lines were exposed to 100 mM of ethanol for 48 h and then phospho-fraction was separated by column chromatography as described in material and methods. The results show that ethanol increased the STARD10 unphosphorylated fraction by 5-fold compared to the control and correspondingly decreased the STARD10 phosphorylated fraction by 90% compared to the control (Fig. 5a). Even though, we found that ethanol has no effects on CKII expression (Fig. 5b and c), its enzymatic activity decreased after ethanol administration by 40 and 20% in MCF-7 and SKBR-3, respectively (Fig. 5d). These results have been confirmed in vivo MMTV-neu transgenic mice tissues (Fig. 5e and f).

### Forced expression of STARD10 and ethanol administration increase membrane fluidity in MCF-7 and SKBR-3 cell lines

**Fig. 6:**
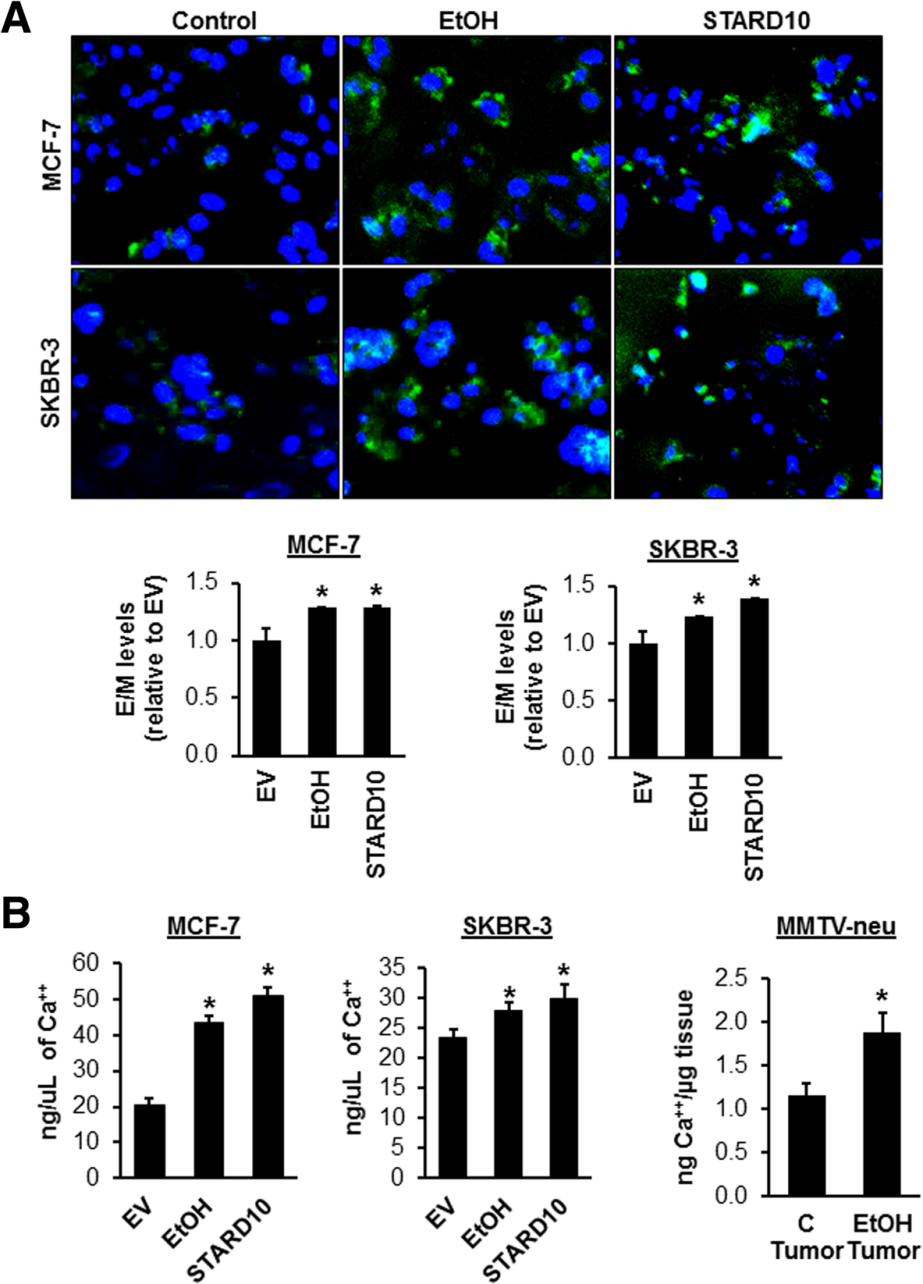
Ethanol and STARD10 overexpression increase membrane fluidity and intracellular calcium concentration in vitro and in vivo. **a** Cells were treated with ethanol (100 mM) for 48 h or transfected with StarD10. Membrane fluidity assay in MCF-7 and SKBR-3 cells. Typical epifluorescence microscopic analysis was performed using an excitation filter (350 nm), a dichroic filter (370 nm) and emission filters for monomer fluorescence (405 nm interference filter) and excimer fluorescence (470 nm cut-on filter). The normalized fluorescence was calculated as a ratio of excimer to monomer. **b** Calcium ion concentrations in cell lysates from ethanol-treated or STARD10 transfected MCF-7 and SKBR-3 cells (100 mM for 48 h). Calcium ion concentration in breast tumor cell lysates from 4 ethanol-fed MMTV-neu transgenic mice. All data are expressed as (mean ± SE) from 3 independent experiments performed in triplicates. *p < 0.05 vs. EV MCF-7 and SKBR-3; *p < 0.04 vs control tumor MMTV-neu

It is well-know that ethanol can influence cell migration and invasion in vitro that modulates cellular viability, proliferation, migration, and invasion in cancer cells [22] [10]. For this reason, membrane fluidity was assayed on live MCF-7 and SKBR-3 cell lines treated with 100 mM ethanol or transiently transfected with STARD10 for 48 h using a fluorescent probe. The use of lipophilic pyrene probes, that undergo excimer formation upon spatial interaction, is considered one of the best systems to study membrane fluidity [32]. Measuring the ratio of excimer (EM 470 nm) to monomer (EM 372) fluorescence, a quantitative monitoring of the membrane fluidity was attained. The confocal microscopy images showed that ethanol increased the membrane fluidity by 1.4-fold in both cell lines (Fig. 6a), and STARD10 forced expression resulted in 1.4- and 1.5-fold increases in fluidity (Fig. 6a lower panel).

### Ethanol and STARD10 mediate calcium transport that increases cytoplasmic calcium concentration

Previous reports have established the fact that increases in cell membrane fluidity cause an increase in calcium ion permeability [33]. For the first time, we confirmed that ethanol administration increases cytoplasmic calcium concentration by 2.2- and 1.2-fold in MCF-7 and SKBR-3 cell lines, respectively (Fig. 6b). Also, we provide evidence that STARD10 overexpression enhanced membrane permeability, leading to increased calcium ion uptake by 2.5- and 1.3-fold in MCF-7 and SKBR-3 cell lines, respectively (Fig. 6b). These results were confirmed in MMTV-neu transgenic mice, that showed a 1.6-fold increase in calcium concentration in the ethanol group compared to control group (Fig. 6b right panel).

### Mechanism of action of ethanol, ERBB2 and STARD10 in breast cancer cell growth and migration

**Fig. 7:**
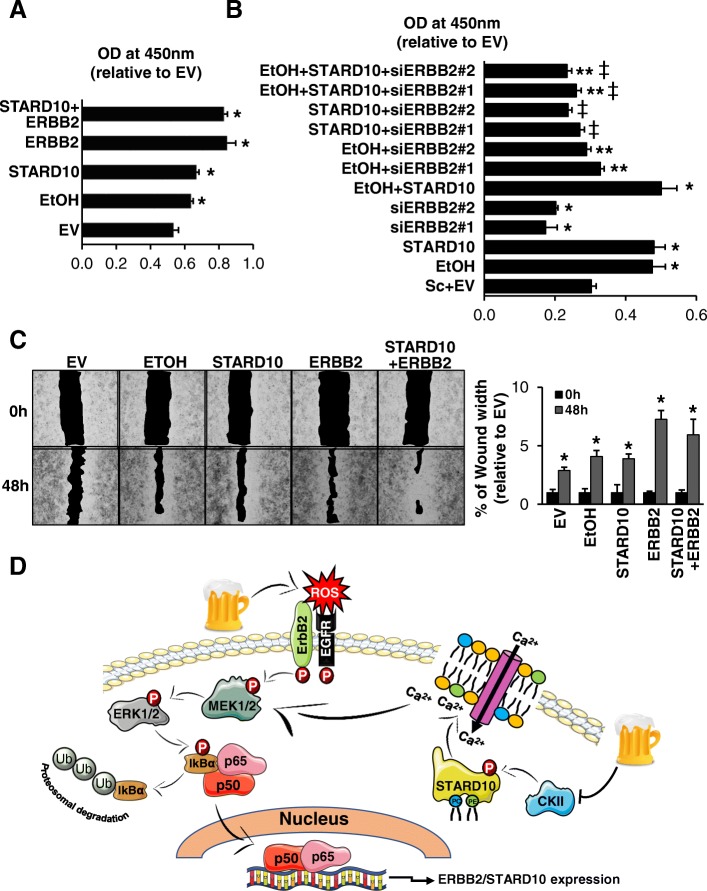
Ethanol administration, STARD10 and ERBB2 overexpression promote breast cancer cell malignancy in MCF-7 cells. MCF-7 cells were treated with 100 mM ethanol and transfected with STARD10 and/or ERBB2 for 48 h (**a**, **b**) MTT assay showing Data are expressed as (mean ± SE) from 3 to 4 independent experiments performed in triplicates. *p < 0.05 vs. EV. **c** Migration assay. Results are shown as total wound area at 0 h and 48 h. Data are expressed as (mean ± SE) from 4 independent experiments performed in triplicates. *p < 0.05 vs. EV 48 h. **d** Pathway schematic depicting known intracellular signaling mechanisms activated downstream of the ethanol administration proposed to mediate cell proliferation as well as cell migration through STARD10 and ERBB2 activation

Several reports demonstrated that ethanol stimulates both cell proliferation and migration of breast cancer cells [10]. Also, increased ERBB2 expression seems to be correlated with the ethanol stimulation [22]. In order to demonstrate that ethanol promotes cell growth and migration via induced-expression of STARD10 and ERBB2, MCF-7 and SKBR3 cell lines were treated for 48 h with 100 mM ethanol. MTT assay was performed to determine the effect of STARD10, ERBB2 and ethanol on cell proliferation, which revealed that STARD10 overexpression enhanced the viability of the mammary tumor cells compared to control in a manner similar to ethanol administration and ERBB2 overexpression (Fig. 7a and Additional file 6: Figure S4B). We also proved by silencing *ERBB2* that it was required for ethanol to sustain the effect on STARD10-mediated cell growth (Fig. 7b and Additional file 6: Figure S4B). Wound-healing assay clearly show that ethanol exposure promoted cell migration by 20% compared to control in both MCF-7 and in SKBR-3 cell lines (Fig. 7c and Additional file 6: Figure S4C). Similarly, the ectopic expression of STARD10 and ERBB2 markedly enhanced the cells’ migration ability compared to the control **(**Figs. 7c and Additional file 6: Figure S4C). STARD10 and ERBB2 co-overexpression caused an induction of migration level, without a corresponding change in viability in both cell lines, compared to single overexpression alone (Fig. 7 and Additional file 6: Figure S4) suggesting us that ERBB2 promoted this migratory event because of STARD10 overexpression. Since STARD10 overexpression induces growth and migration (Additional file 6: Figure S4), we investigated whether silencing STARD10 could have the reverse effect on these parameters. The efficiency of STARD10 siRNA as assessed by qRTPCR was higher for siRNA#1 compared to siRNA#2 **(**Additional file 7: Figure S5A and S5D). Surprisingly, we found that similar to STARD10 overexpression, its silencing also induced the growth rate of MCF-7 and SKBR3 cells compared to control siRNA (Additional file 7: Figure S5B and S5E). Similar to the overexpression results, siSTARD10 also induced migration capability of MCF-7 and SKBR-3 cells (Additional file 7: Figure S5C and S5F). The results suggest that a balanced level of STARD10 is important for regulating the proliferative activity in breast cancer and its dysregulation in either direction (increase or decrease) leads to an increase in cell proliferation and migration with consequent increase in neoplastic progression. The findings and proposed scheme of events are summarized in Fig. 7d. Surprisingly the results indicated that inhibition of STARD10 significantly increased the growth rate of both cell lines compared to scramble siRNA (Additional file 7: Figure S5B and S5E). Similar results were observed in migration capability of MCF-7 and SKBR-3 cells (Additional file 7: Figure S5C and S5F). The results suggest that the steadiness of STARD10 is important for regulating the proliferative activity in breast cancer and its dysregulation leads to an increase in cell proliferation and migration with consequent increase in neoplastic progression. The findings and proposed scheme of events are summarized in Fig. 7d.

## Discussion

Alcohol abuse has been reported to promote mammary tumorigenesis enhancing cell growth in vitro and in vivo [34, 22]*.* In addition to its carcinogenic effect, alcohol abuse is associated with progression and aggressiveness of existing mammary tumors [35]. Mammary tissues and breast cancer cells normally metabolize alcohol by CYP2E1, ADH, xanthine oxidoreductase (XOR), and NOX which produces ROS, causing oxidative stress [11, 36, 37]*.* Specifically, CYP2E1 is one of the most active ROS-generating CYP450 isoforms and it is considered the link between oxidative stress and tumor growth. In addition, CYP2E1 expression in breast cancer cells plays a role in the migratory capacity, autophagy, ER stress and metastasis [11].

Human breast cancer cells or mammary epithelial cells with a high expression of receptor tyrosine-protein kinase ERBB2 exhibited an enhanced response to ethanol-stimulated cell invasion in vitro [22]*,* therefore ethanol stimulates ROS production in mammary epithelial cells in an ERBB2-dependent manner [38]. ERBB2 belongs to the epidermal growth factor receptor (EGFR) family and plays an important role in cell proliferation and transformation through formation of heterodimers with EGFR and HER3 [39]*.* No known ligand has been identified for ERBB2, ethanol induces its phosphorylation that activates the mitogen-activated protein kinase MAPK signaling members, extracellular signal-regulated kinase ERK and other several important signaling cascades well-known to be downstream target of ERBB2 that play a key role in the carcinogenesis and aggressiveness of breast cancer [40]*.* STARD10 is a specific lipid carrier for PC and PE, is well-known to be overexpressed in Neu/ErbB2-induced mammary tumors in transgenic mice, in several human breast carcinoma cell lines, and in 35% of primary human breast cancers [16]*.* It was found to be co-expressed with ERBB2 in Neu tumors and human breast carcinoma cell lines and was demonstrated to cooperate with ErbB pathway in cellular transformation [20]*.* In this paper we tried to elucidate the mechanism by which ERBB2/STARD10 crosstalk promotes ethanol induced cell growth and migration in breast cancer cells. We also provide evidence that the common transcription factor p65 is involved in mediating co-expression of STARD10 and ERBB2. Our results indicate a mutual induction of STARD10 and ERBB2 that positively regulates ethanol-induced malignancy/aggressiveness phenotype. This is supported by the finding that MCF-7 and SKBR-3 cell lines are more susceptible to cell growth and migration when treated with ethanol, which induces both STARD10 and ERBB2 and also overexpressing these key players. In resting cells, NF-kB is cytoplasmic sequestered as a latent complex bound to one or more members of the IkB protein family (IkBa, IkBb, IkBe, IkBg). Ethanol stimuli through ERBB2 phosphorylation activates the mitogen activated protein kinase (MAPK) signaling members than induce phosphorylation via activation of the IkB kinase complex, IKK) and subsequent proteasomal degradation of IkB inhibitory proteins, activating NF-kB for nuclear translocation. In the nucleus the p65/p50 heterodimer binds *ERBB2* promoter-specific consensus DNA elements [28] and for the first time we provide evidence that p65 also binds to *STARD10* promoter positively regulating its transcription. STARD10 transfers PC and PE between membranes, replenishing membranes with lipids metabolized by phospholipases. Lipids are delivered via monomeric exchange between the cytosolic membrane surfaces of different organelles. Monomeric exchange requires desorption of the lipid from the donor membrane, passage through the aqueous phase, and subsequent insertion into the acceptor membrane [41]*.* This is the first report demonstrating that the increased STARD10 protein amount can change the membrane fluidity with a consequent increase in membrane permeability to calcium ions (Ca^2+^). It is well known that elevated intracellular Ca^2+^ triggers numerous signaling pathways including protein kinases such as the calmodulin-dependent kinases (CaMKs) and the extra-cellular signal-regulated kinases (ERKs) [42]*.* These results support a novel hypothesis that a key mechanism for ethanol-induced STARD10 to promote ERBB2 is via its function as a lipid transporter.

## Conclusions

In summary, the data presented in this study clearly showed that the ability of STARD10 to influence ERBB2 expression and activity may be involve both dependent and independent lipid binding function. This is the first report demonstrating that ethanol can modulate in dynamic manner the ERBB2 role through STARD10 involvement in breast cancer.

## Additional files

Additional file 1: **Table S1.** Characteristics of breast cancer tissues from ErbB2-positive patients. (DOCX 358 kb)

Additional file 2: **Table S2.** STARD10 expression in human breast cancer databases. Biological replicates (Rep.) are parallel measurements of biologically distinct samples that capture random biological variation. (DOCX 358 kb)

Additional file 3: **Figure S1.** Ethanol-induced p65 that increaseS STARD10 and ERBB2 expression in SKBR-3 cells. **(A)** SKBR-3 cells were treated with 100 mM ethanol and transfected with STARD10 and/or ERBB2 for 48 h. STARD10 and ERBB2 mRNA and protein levels were accomplished using RT-PCR and Western blot analysis, respectively, compared to control from 4 independent experiments. *p < 0.05 vs. EV. **(B)** mRNA levels of STARD10 and ERBB2 in SKBR-3 cells treated with ethanol (100 mM) and transfected with STARD10 overexpression vector and ERBB2 siRNA (10 nM) for 48 h. Results are expressed as fold relative to Sc + EV (mean ± SE) from 3 independent experiments. *p < 0.02 vs.Sc + EV; †*p* < 0.01 vs. STARD10; ‡*p* < 0.05 vs. EtOH. **(C)** *STARD10* and *ERBB2* promoter activity analysis was performed using reporter assay from 4 independent experiments. *p < 0.02 vs. EV STARD10 promoter; *p < 0.04 vs. EV ERBB2 promoter. (D) Cells were treated with 100 mM ethanol or transfected with p65 plasmid for 48 h. STARD10 and ERBB2 expression was analyzed by RT-PCR and Western Blotting analysis to measure their mRNA and protein levels. Results are expressed as fold relative to EV (mean ± SE) from 3 independent experiments. *p < 0.05 vs. EV mRNA; *p < 0.03 vs. EV proteins. (PPTX 69 kb)

Additional file 4: **Figure S2.** ERBB2 and its downstream targets overexpression positively regulate STARD10 expression in MCF-7 cells. Cell were treated with 100 mM ethanol for 48 h. **(A)** ERBB2 downstream targets binding sites on human *STARD10* promoter sequence. **(B)** *STARD10* promoter activity by reporter assay. **(C)** RT-PCR of StarD10 mRNA level. Results are expressed as percentage relative to EV for promoter analysis and as fold relative to EV for mRNA level. Statistically significant in four independent experiments. *p < 0.04 vs EV promoter; *p < 0.05 vs EV mRNA. **(D)** Protein levels were examined by Western blotting using an anti-STARD10 antibody. Results were expressed as fold relative to EV. Data are expressed as (mean ± SE) from triplicate of four independent experiments. *p < 0.05 vs. EV. (PPTX 600 kb)

Additional file 5: **Figure S3.** p65 positively regulates ethanol-induced STARD10 and ERBB2 expression binding their promoter sequence in SKBR-3 cells. Cells were treated with 100 mM ethanol and transfected with p65 siRNA (10 nM) for 48 h (**A)** *STARD10* promoter activity assay. *p < 0.003 vs. Sc. †*p* < 0.003 vs. EtOH. ERBB2 promoter activity assay. *p < 0.04 vs. Sc. †*p* < 0.04 vs. EtOH. **(B**) Relative expression of *STARD10*, *ERBB2*, and *RELA* mRNA and the efficiency p65 silencing were determined by qRT-PCR; *p < 0.05 vs. Sc. †*p* < 0.0003 vs. EtOH. **(C)** Nuclear and cytoplasmid p65 protein level were analyzed by Western blotting. Nuclear marker (H3) and cytosolic marker (tubulin) were immunoblotted to demonstrate fraction purity. Data are expressed as (mean ± SE) from triplicate of four independent experiments. *p < 0.05 vs. control. (PPTX 1155 kb)

Additional file 6: **Figure S4.** Ethanol administration, STARD10 and ERBB2 overexpression promote cell malignancy in SKBR-3 cells. Cells were treated with 100 mM ethanol and transfected with STARD10 and ERBB2 plasmids or *ERBB2* siRNA (10 nM)for 48 h **(A) (B)** MTT assay. Data are expressed as (mean ± SE) from 4 to 5 independent experiments performed in triplicates. *p < 0.04 vs. EV; *p < 0.01 vs. Sc + EV; †*p* < 0.02 vs. EtOH; ‡*p* < 0.02 vs. STARD10. **(C)** Migration assay. Results are shown as total wound area at 0 h and 48 h. Data are expressed as (mean ± SE) from 5 independent experiments performed in triplicates. *p < 0.05 vs. EV 48 h. (PPTX 600 kb)

Additional file 7: **Figure S5**. STARD10 silencing increases cell malignancy in vitro. MCF-7 and SKBR-3 cells were transfected with two different *STARD10* siRNAs (10 nM) for 48 h. **(A)(D)** Efficiency of *STARD10* silencing was determined by qRT-PCR. Data are expressed as (mean ± SE) from 3 independent experiments performed in triplicates.*p < 0.001 vs. Sc MCF-7 cells; *p < 0.001 vs. Sc SKBR-3 cells **(B)(E)** MTT assay showing viability of MCF-7 cells transfected with two different StarD10 silencers. Data are expressed as (mean ± SE) from 3 independent experiments performed in triplicates. *p < 0.04 vs. Sc MCF-7 cells; *p < 0.03 vs. Sc SKBR-3 cells. **(C)(F)** Graphs of migration assay. Data are expressed as (mean ± SE) from 4 independent experiments performed in triplicates. 48 h. Results are shown as percent of wound with compared to Sc at 0 h and 48 h. Data are expressed as (mean ± SE) from 3 independent experiments performed in triplicates. *p < 0.04 vs. Sc 0 h MCF-7 cells; †*p* < 0.05 vs. Sc 48 h MCF-7 cells. *p < 0.05 vs. Sc 0 h; †*P* < 0.05 vs. Sc 48 h SKBR-3 cells. (PPTX 112 kb)

## Abbreviations

Akt: AK strain transforming
Ca2+: calcium ions
ChIP: Chromatin immunoprecipitation
CKII: Casein Kinase II
ERBB2: Receptor tyrosine-protein kinase
ERK: Extracellular signal-regulated kinase
EtOH: Ethanol
JNK1/2: c-Jun NH2 terminal protein kinase
MAPKs: Signaling members mitogen-activated protein kinases
MMTV-neu: Mouse mammary tumor virus
MTT: 3-(4,5-dimethylthiazol-2-yl)-2,5-diphenyltetrazolium bromide
p38 MAPK: p38 mitogen-activated protein kinase
PC: Phosphatidylcholine
PE: Phosphatidylethanolamine
PI3-kinase: Phosphatidyl inositol 3 kinase
STARD10: StAR-related lipid transfer protein 10
