# Supplementary material for: Star-related lipid transfer protein 10 (STARD10): a novel key player in alcohol-induced breast cancer progression
Source: J Exp Clin Cancer Res. 2019 Jan 5;38:4. doi: 10.1186/s13046-018-1013-y (PMC6321732; doi:10.1186/s13046-018-1013-y)
Supplement: Supplementary file 2 — Table S2. STARD10 expression in human breast cancer databases. Biological replicates (Rep.) are parallel measurements of biologically distinct samples that capture random biological variation. (DOCX 358 kb) [file 13046_2018_1013_MOESM2_ESM.docx]

| **Sample** | **Title** | **Value** | **Rank** | **x value** | **value** | **median** | **ttest** |
| --- | --- | --- | --- | --- | --- | --- | --- |
| **GSM535613** | healthy breast 1, biological rep. 1 | 36.844 | 63 | 1.01 | 36.844 | 111.81 |  |
| **GSM535614** | healthy breast 2, biological rep. 2 | 133.67 | 75 | 1.02 | 133.67 |  |  |
| **GSM535615** | healthy breast 3, biological rep. 3 | 123.11 | 74 | 0.99 | 123.11 |  |  |
| **GSM535616** | healthy breast 4, biological rep. 4 | 40.552 | 64 | 0.98 | 40.552 |  |  |
| **GSM535617** | healthy breast 5, biological rep. 5 | 100.52 | 73 | 0.97 | 100.52 |  |  |
| **GSM535613** | healthy breast 1, biological rep. 1 | 33.46 | 62 | 0.96 | 33.46 |  |  |
| **GSM535614** | healthy breast 2, biological rep. 2 | 154.74 | 77 | 1.15 | 154.74 |  |  |
| **GSM535615** | healthy breast 3, biological rep. 3 | 160.01 | 77 | 1.21 | 160.01 |  |  |
| **GSM535616** | healthy breast 4, biological rep. 4 | 85.25 | 71 | 1.03 | 85.25 |  |  |
| **GSM535617** | healthy breast 5, biological rep. 5 | 180.94 | 79 | 1.09 | 180.94 |  |  |
| **GSM535604** | DCIS 1, biological rep. 1 | 94.224 | 71 | 3.51 | 94.224 | 415.01 | 0.0095294 |
| **GSM535605** | DCIS 2, biological rep. 2 | 355.97 | 86 | 3.65 | 355.97 |  |  |
| **GSM535606** | DCIS 3, biological rep. 3 | 560.8 | 91 | 3.61 | 560.8 |  |  |
| **GSM535607** | DCIS 4, biological rep. 4 | 137.59 | 75 | 3.52 | 137.59 |  |  |
| **GSM535608** | DCIS 5, biological rep. 5 | 1382.6 | 96 | 3.25 | 1382.6 |  |  |
| **GSM535609** | DCIS 6, biological rep. 6 | 318 | 85 | 3.33 | 318 |  |  |
| **GSM535610** | DCIS 7, biological rep. 7 | 384.32 | 87 | 3.6 | 384.32 |  |  |
| **GSM535611** | DCIS 8, biological rep. 8 | 479.93 | 89 | 3.35 | 479.93 |  |  |
| **GSM535612** | DCIS 9, biological rep. 9 | 90.014 | 71 | 3.39 | 90.014 |  |  |
| **GSM535604** | DCIS 1, biological rep. 1 | 184.14 | 79 | 3.62 | 184.14 |  |  |
| **GSM535605** | DCIS 2, biological rep. 2 | 501.82 | 89 | 3.39 | 501.82 |  |  |
| **GSM535606** | DCIS 3, biological rep. 3 | 698.15 | 92 | 3.54 | 698.15 |  |  |
| **GSM535607** | DCIS 4, biological rep. 4 | 143.77 | 76 | 3.21 | 143.77 |  |  |
| **GSM535608** | DCIS 5, biological rep. 5 | 1702.6 | 96 | 3.98 | 1702.6 |  |  |
| **GSM535609** | DCIS 6, biological rep. 6 | 445.71 | 88 | 3.87 | 445.71 |  |  |
| **GSM535610** | DCIS 7, biological rep. 7 | 517.82 | 89 | 3.67 | 517.82 |  |  |
| **GSM535611** | DCIS 8, biological rep. 8 | 732.75 | 92 | 3.99 | 732.75 |  |  |
| **GSM535612** | DCIS 9, biological rep. 9 | 158.52 | 78 | 3.97 | 158.52 |  |  |
| **GSM535618** | IDC 1, biological rep. 1 | 915.54 | 94 | 6.2 | 915.54 | 961.98 | 1.624E-08 |
| **GSM535619** | IDC 2, biological rep. 2 | 1218.2 | 95 | 6.09 | 1218.2 |  |  |
| **GSM535620** | IDC 3, biological rep. 3 | 967.15 | 94 | 6.15 | 967.15 |  |  |
| **GSM535621** | IDC 4, biological rep. 4 | 1360 | 96 | 6.1 | 1360 |  |  |
| **GSM535622** | IDC 5, biological rep. 5 | 611.36 | 91 | 6.05 | 611.36 |  |  |
| **GSM535618** | IDC 1, biological rep. 1 | 956.81 | 94 | 6.01 | 956.81 |  |  |
| **GSM535619** | IDC 2, biological rep. 2 | 1035.7 | 94 | 6.03 | 1035.7 |  |  |
| **GSM535620** | IDC 3, biological rep. 3 | 793.12 | 93 | 6.12 | 793.12 |  |  |
| **GSM535621** | IDC 4, biological rep. 4 | 1190.8 | 95 | 6.06 | 1190.8 |  |  |
| **GSM535622** | IDC 5, biological rep. 5 | 474.61 | 89 | 6.21 | 474.61 |  |  |

**Table S2.** STARD10 expression in human breast cancer databases. Biological replicates (Rep.) are parallel measurements of biologically distinct samples that capture random biological variation.
