# Supplementary material for: Star-related lipid transfer protein 10 (STARD10): a novel key player in alcohol-induced breast cancer progression
Source: J Exp Clin Cancer Res. 2019 Jan 5;38:4. doi: 10.1186/s13046-018-1013-y (PMC6321732; doi:10.1186/s13046-018-1013-y)
Supplement: Supplementary file 3 — Figure S1. Ethanol-induced p65 that increaseS STARD10 and ERBB2 expression in SKBR-3 cells. (A) SKBR-3 cells were treated with 100 mM ethanol and transfected with STARD10 and/or ERBB2 for 48 h. STARD10 and ERBB2 mRNA and protein levels were accomplished using RT-PCR and Western blot analysis, respectively, compared to control from 4 independent experiments. *p < 0.05 vs. EV. (B) mRNA levels of STARD10 and ERBB2 in SKBR-3 cells treated with ethanol (100 mM) and transfected with STARD10 overexpression vector and ERBB2 siRNA (10 nM) for 48 h. Results are expressed as fold relative to Sc + EV (mean ± SE) from 3 independent experiments. *p < 0.02 vs.Sc + EV; †p < 0.01 vs. STARD10; ‡p < 0.05 vs. EtOH. (C) STARD10 and ERBB2 promoter activity analysis was performed using reporter assay from 4 independent experiments. *p < 0.02 vs. EV STARD10 promoter; *p < 0.04 vs. EV ERBB2 promoter. (D) Cells were treated with 100 mM ethanol or transfected with p65 plasmid for 48 h. STARD10 and ERBB2 expression was analyzed by RT-PCR and Western Blotting analysis to measure their mRNA and protein levels. Results are expressed as fold relative to EV (mean ± SE) from 3 independent experiments. *p < 0.05 vs. EV mRNA; *p < 0.03 vs. EV proteins. (PPTX 69 kb) [file 13046_2018_1013_MOESM3_ESM.pptx]

## Slide 1
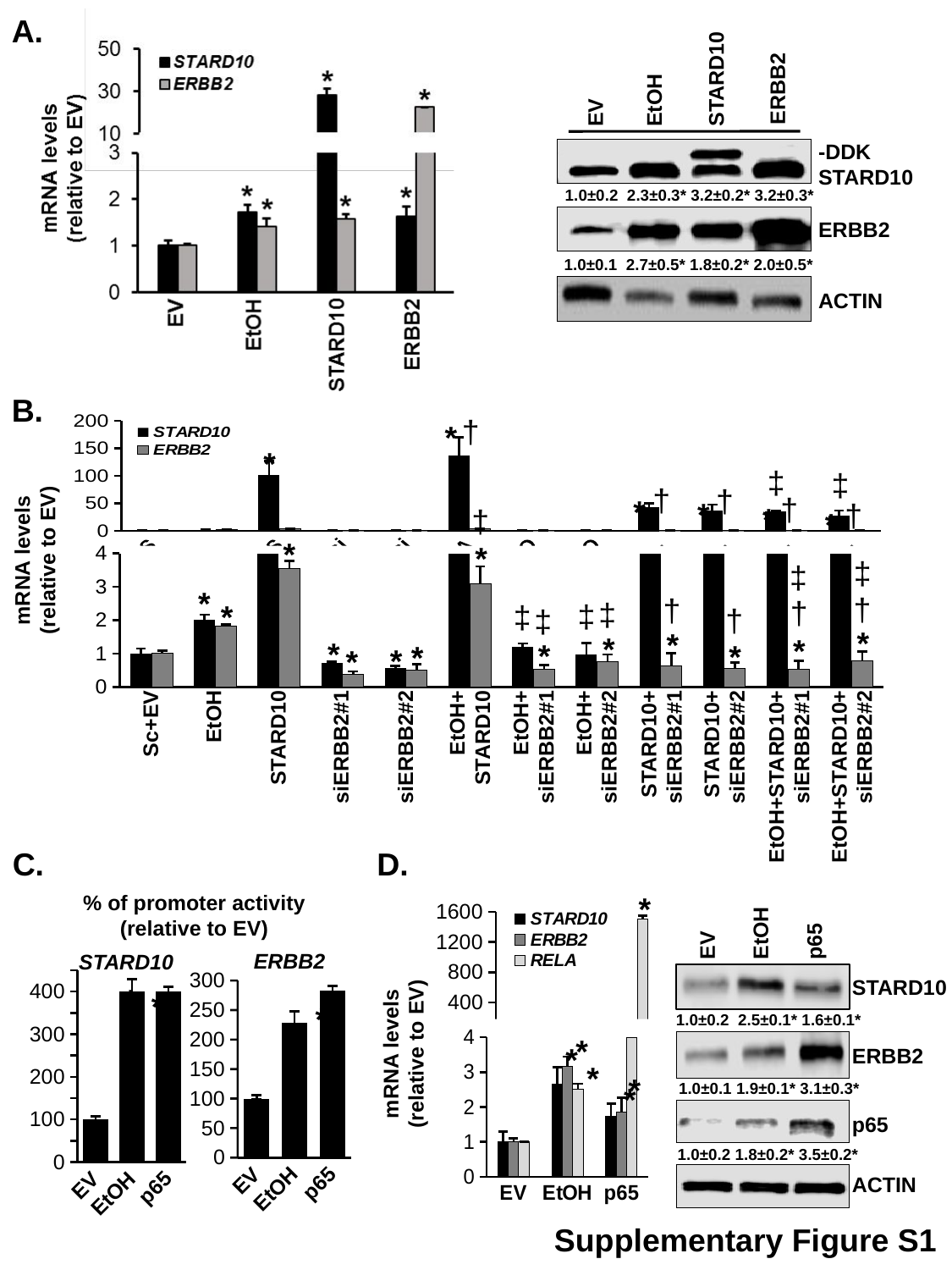

A.
ERBB2
STARD10
EV
EtOH
-DDK
STARD10
1.0±0.2 2.3±0.3* 3.2±0.2* 3.2±0.3*
ERBB2
1.0±0.1 2.7±0.5* 1.8±0.2* 2.0±0.5*
ACTIN
B.
†
*
### Chart
| Category | STARD10 | ERBB2 |
|---|---|---|
| Sc+EV | 1.0 | 1.0 |
| EtOH | 2.0031626961508007 | 1.8314512686341047 |
| STARD10 | 101.74466348498532 | 3.549966541259529 |
| siERBB2#1 | 0.715462768714684 | 0.3843470233549004 |
| siERBB2#2 | 0.5666728113416635 | 0.4979199546792655 |
| STARD10+EtOH | 136.72584363210441 | 3.0859268227845655 |
| EtOH+siERBB2#1 | 1.203536254381102 | 0.5260395433613918 |
| EtOH+siERBB2#2 | 0.9698305585339274 | 0.747848708654275 |
| STARD10+siERBB2#1 | 42.45273034980252 | 0.6212086711736895 |
| STARD10+siERBB2#2 | 37.38276440776358 | 0.5496850736421347 |
| EtOH+STARD10+siERBB2#1 | 34.727034540641 | 0.5365265198728539 |
| EtOH+STARD10+siERBB2#2 | 27.012999571987926 | 0.7811218819023804 |
### Chart
| Category | STARD10 | ERBB2 |
|---|---|---|
| Sc+EV | 1.0 | 1.0 |
| EtOH | 2.0031626961508007 | 1.8314512686341047 |
| STARD10 | 101.74466348498532 | 3.549966541259529 |
| siERBB2#1 | 0.715462768714684 | 0.3843470233549004 |
| siERBB2#2 | 0.5666728113416635 | 0.4979199546792655 |
| STARD10+EtOH | 136.72584363210441 | 3.0859268227845655 |
| EtOH+siERBB2#1 | 1.203536254381102 | 0.5260395433613918 |
| EtOH+siERBB2#2 | 0.9698305585339274 | 0.747848708654275 |
| STARD10+siERBB2#1 | 42.45273034980252 | 0.6212086711736895 |
| STARD10+siERBB2#2 | 37.38276440776358 | 0.5496850736421347 |
| EtOH+STARD10+siERBB2#1 | 34.727034540641 | 0.5365265198728539 |
| EtOH+STARD10+siERBB2#2 | 27.012999571987926 | 0.7811218819023804 |*
‡
‡
†
†
†
*
*
†
*
†
*
*
*
mRNA levels
(relative to EV)
‡
‡
*
†
†
†
‡
‡
‡
*
†
‡
*
*
*
*
*
*
*
*
*
*
EtOH+ STARD10
Sc+EV
EtOH
STARD10
EtOH+
siERBB2#1
EtOH+
siERBB2#2
STARD10+
siERBB2#1
STARD10+
siERBB2#2
siERBB2#1
siERBB2#2
EtOH+STARD10+
siERBB2#1
EtOH+STARD10+
siERBB2#2
C.
D.
EV
EtOH
p65
1.0±0.2 2.5±0.1* 1.6±0.1*
1.0±0.1 1.9±0.1* 3.1±0.3*
1.0±0.2 1.8±0.2* 3.5±0.2*
ACTIN
STARD10
ERBB2
p65
*
### Chart
| Category | STARD10 | ERBB2 | RELA |
|---|---|---|---|
| EV | 1.0 | 1.0 | 1.0 |
| EtOH | 2.6544198283655116 | 3.168000616814442 | 2.5 |
| p65 | 1.727450943545831 | 1.8641823900315593 | 1501.7635 |
### Chart
| Category | STARD10 | ERBB2 | RELA |
|---|---|---|---|
| EV | 1.0 | 1.0 | 1.0 |
| EtOH | 2.6544198283655116 | 3.168000616814442 | 2.5 |
| p65 | 1.727450943545831 | 1.8641823900315593 | 1501.7635 |mRNA levels
(relative to EV)
*
*
*
*
*
% of promoter activity
(relative to EV)
### Chart
| Category | |
|---|---|
| EV | 100.0 |
| EtOH | 228.6983434334279 |
| p65 | 283.50274571414616 |*
*
ERBB2
STARD10
### Chart
| Category | |
|---|---|
| EV | 100.0 |
| EtOH | 398.4029320189181 |
| p65 | 400.3334651169527 |*
*
Supplementary Figure S1
