# Supplementary material for: Star-related lipid transfer protein 10 (STARD10): a novel key player in alcohol-induced breast cancer progression
Source: J Exp Clin Cancer Res. 2019 Jan 5;38:4. doi: 10.1186/s13046-018-1013-y (PMC6321732; doi:10.1186/s13046-018-1013-y)
Supplement: Supplementary file 4 — Figure S2. ERBB2 and its downstream targets overexpression positively regulate STARD10 expression in MCF-7 cells. Cell were treated with 100 mM ethanol for 48 h. (A) ERBB2 downstream targets binding sites on human STARD10 promoter sequence. (B) STARD10 promoter activity by reporter assay. (C) RT-PCR of StarD10 mRNA level. Results are expressed as percentage relative to EV for promoter analysis and as fold relative to EV for mRNA level. Statistically significant in four independent experiments. *p < 0.04 vs EV promoter; *p < 0.05 vs EV mRNA. (D) Protein levels were examined by Western blotting using an anti-STARD10 antibody. Results were expressed as fold relative to EV. Data are expressed as (mean ± SE) from triplicate of four independent experiments. *p < 0.05 vs. EV. (PPTX 600 kb) [file 13046_2018_1013_MOESM4_ESM.pptx]

## Slide 1
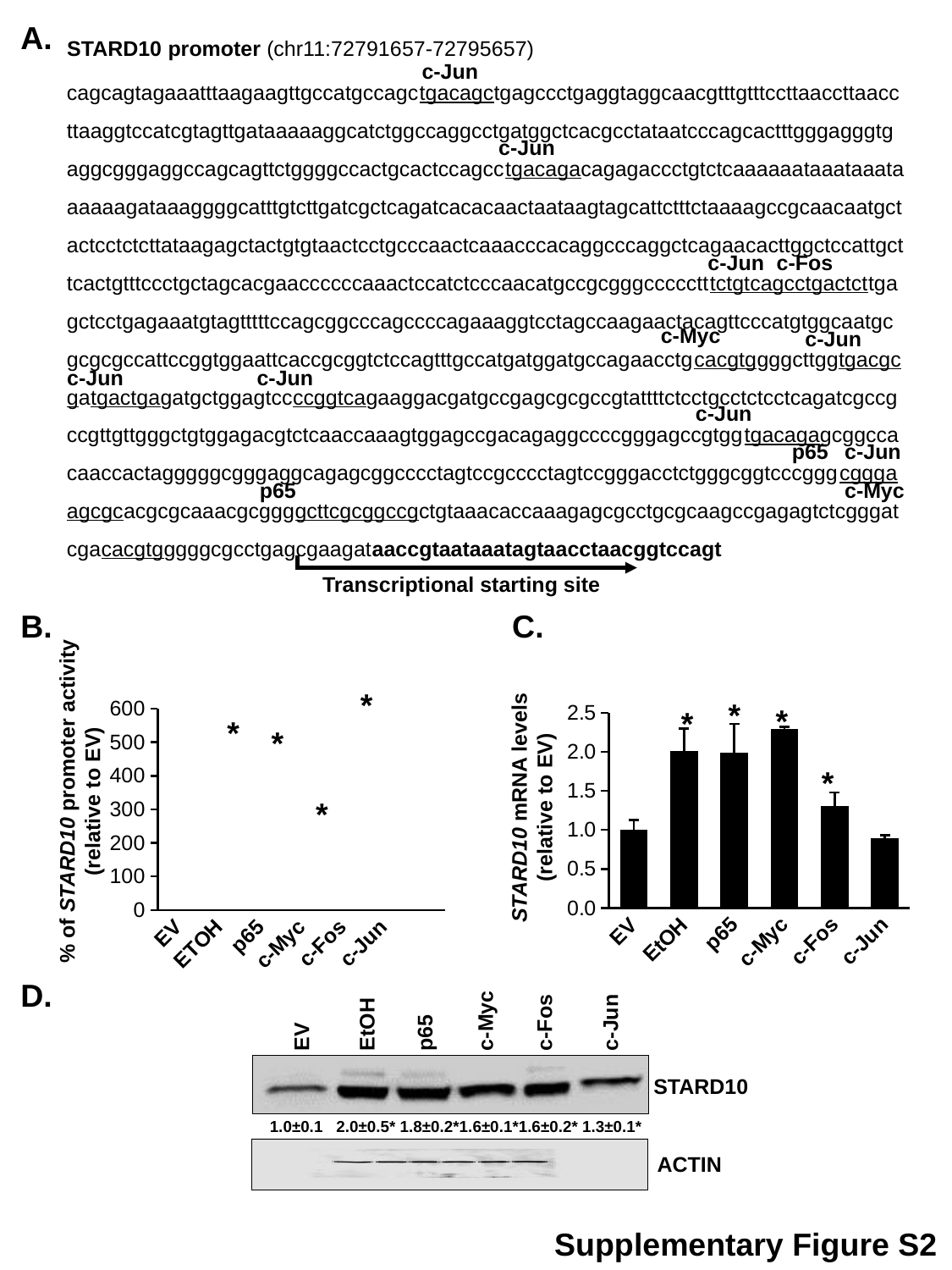

STARD10 promoter (chr11:72791657-72795657)
c-Jun
cagcagtagaaatttaagaagttgccatgccagctgacagctgagccctgaggtaggcaacgtttgtttccttaaccttaaccttaaggtccatcgtagttgataaaaaggcatctggccaggcctgatggctcacgcctataatcccagcactttgggagggtgaggcgggaggccagcagttctggggccactgcactccagcctgacagacagagaccctgtctcaaaaaataaataaataaaaaagataaaggggcatttgtcttgatcgctcagatcacacaactaataagtagcattctttctaaaagccgcaacaatgctactcctctcttataagagctactgtgtaactcctgcccaactcaaacccacaggcccaggctcagaacacttggctccattgcttcactgtttccctgctagcacgaaccccccaaactccatctcccaacatgccgcgggccccctttctgtcagcctgactcttgagctcctgagaaatgtagtttttccagcggcccagccccagaaaggtcctagccaagaactacagttcccatgtggcaatgcgcgcgccattccggtggaattcaccgcggtctccagtttgccatgatggatgccagaacctgcacgtggggcttggtgacgcgatgactgagatgctggagtccccggtcagaaggacgatgccgagcgcgccgtattttctcctgcctctcctcagatcgccgccgttgttgggctgtggagacgtctcaaccaaagtggagccgacagaggccccgggagccgtggtgacagagcggccacaaccactagggggcgggaggcagagcggcccctagtccgcccctagtccgggacctctgggcggtcccgggcgggaagcgcacgcgcaaacgcggggcttcgcggccgctgtaaacaccaaagagcgcctgcgcaagccgagagtctcgggatcgacacgtgggggcgcctgagcgaagataaccgtaataaatagtaacctaacggtccagt
c-Jun
c-Jun
c-Fos
c-Myc
c-Jun
c-Jun
c-Jun
c-Jun
c-Jun
p65
p65
c-Myc
Transcriptional starting site
A.
B.
C.
*
### Chart
| Category | |
|---|---|
| EV | 100.0 |
| ETOH | 430.95357064493925 |
| p65 | 407.97481869254784 |
| c-Myc | 232.76010886654237 |
| c-Fos | 502.79023987118046 |
| c-Jun | 145.67324338990494 |*
*
% of STARD10 promoter activity
(relative to EV)
*
*
### Chart
| Category | StarD10 |
|---|---|
| EV | 1.0 |
| EtOH | 2.01 |
| p65 | 1.99 |
| c-Myc | 2.29 |
| c-Fos | 1.31 |
| c-Jun | 0.89 |*
*
*
STARD10 mRNA levels
(relative to EV)
D.
EV
EtOH
p65
c-Myc
c-Fos
c-Jun
STARD10
ACTIN
1.0±0.1 2.0±0.5* 1.8±0.2*1.6±0.1*1.6±0.2* 1.3±0.1*
Supplementary Figure S2
