# Supplementary material for: Star-related lipid transfer protein 10 (STARD10): a novel key player in alcohol-induced breast cancer progression
Source: J Exp Clin Cancer Res. 2019 Jan 5;38:4. doi: 10.1186/s13046-018-1013-y (PMC6321732; doi:10.1186/s13046-018-1013-y)
Supplement: Supplementary file 5 — Figure S3. p65 positively regulates ethanol-induced STARD10 and ERBB2 expression binding their promoter sequence in SKBR-3 cells. Cells were treated with 100 mM ethanol and transfected with p65 siRNA (10 nM) for 48 h (A) STARD10 promoter activity assay. *p < 0.003 vs. Sc. †p < 0.003 vs. EtOH. ERBB2 promoter activity assay. *p < 0.04 vs. Sc. †p < 0.04 vs. EtOH. (B) Relative expression of STARD10, ERBB2, and RELA mRNA and the efficiency p65 silencing were determined by qRT-PCR; *p < 0.05 vs. Sc. †p < 0.0003 vs. EtOH. (C) Nuclear and cytoplasmid p65 protein level were analyzed by Western blotting. Nuclear marker (H3) and cytosolic marker (tubulin) were immunoblotted to demonstrate fraction purity. Data are expressed as (mean ± SE) from triplicate of four independent experiments. *p < 0.05 vs. control. (PPTX 1155 kb) [file 13046_2018_1013_MOESM5_ESM.pptx]

## Slide 1
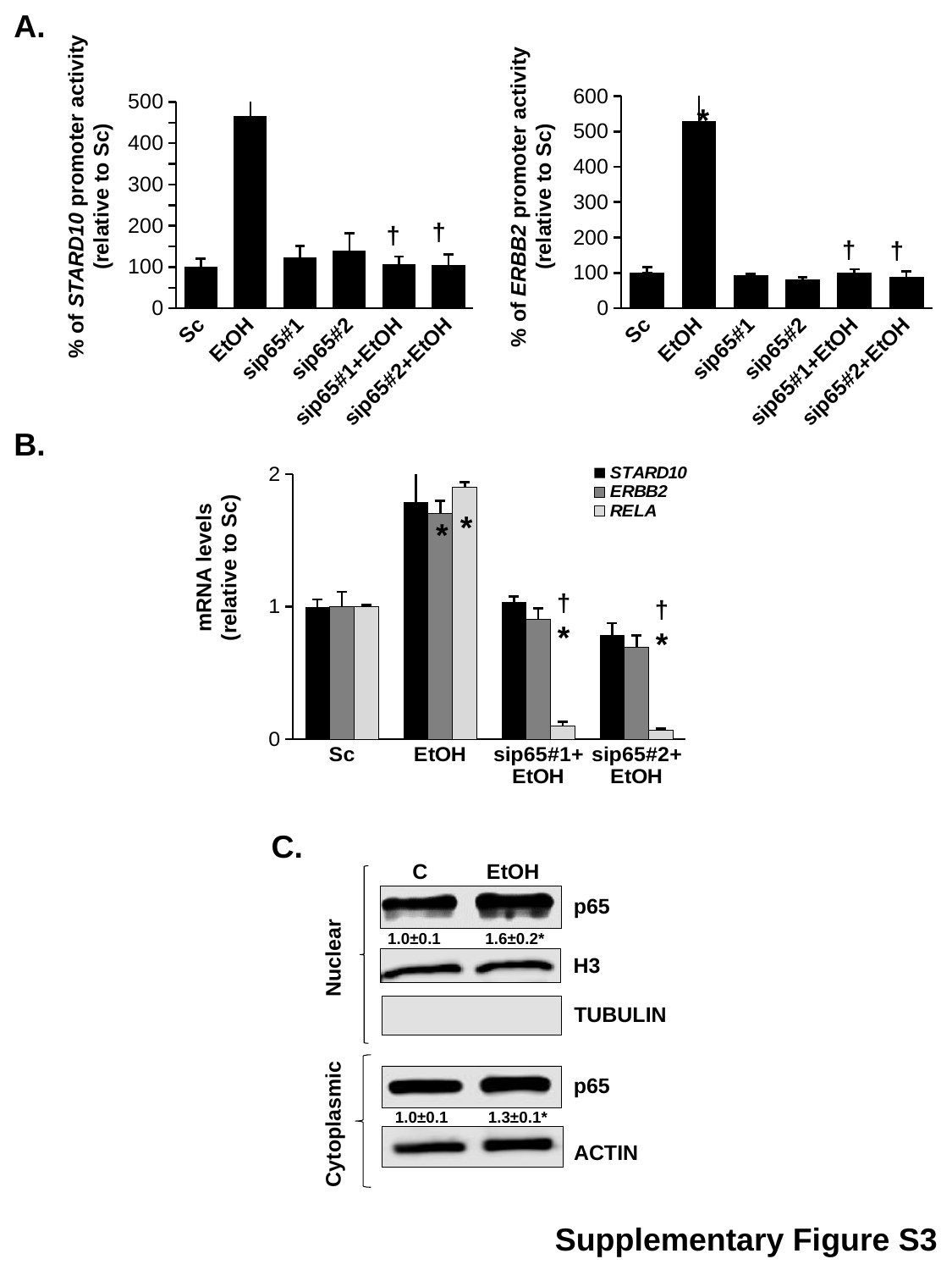

A.
### Chart
| Category | |
|---|---|
| Sc | 100.0 |
| EtOH | 530.3501907737149 |
| sip65#1 | 94.18830245153714 |
| sip65#2 | 80.47453555668686 |
| sip65#1+EtOH | 99.85234309596296 |
| sip65#2+EtOH | 89.28037587824367 |
### Chart
| Category | |
|---|---|
| Sc | 100.0 |
| EtOH | 465.48108770925 |
| sip65#1 | 124.34566885982701 |
| sip65#2 | 139.01476764934478 |
| sip65#1+EtOH | 106.85554270191913 |
| sip65#2+EtOH | 104.09886592096773 |*
*
% of ERBB2 promoter activity
(relative to Sc)
% of STARD10 promoter activity
(relative to Sc)
†
†
†
†
B.
### Chart
| Category | STARD10 | ERBB2 | RELA |
|---|---|---|---|
| Sc | 1.0 | 1.0 | 1.0 |
| EtOH | 1.7903843942270716 | 1.7045239852425202 | 1.901457719021205 |
| sip65#1+EtOH | 1.035264923841378 | 0.9027010292788907 | 0.0970586093751301 |
| sip65#2+EtOH | 0.7873079765692 | 0.6944835426630585 | 0.07124894251221146 |*
*
*
mRNA levels
(relative to Sc)
†
†
*
*
C.
C EtOH
p65
 1.0±0.1 1.6±0.2*
Nuclear
H3
TUBULIN
p65
Cytoplasmic
ACTIN
 1.0±0.1 1.3±0.1*
Supplementary Figure S3
