# Supplementary material for: Star-related lipid transfer protein 10 (STARD10): a novel key player in alcohol-induced breast cancer progression
Source: J Exp Clin Cancer Res. 2019 Jan 5;38:4. doi: 10.1186/s13046-018-1013-y (PMC6321732; doi:10.1186/s13046-018-1013-y)
Supplement: Supplementary file 6 — Figure S4. Ethanol administration, STARD10 and ERBB2 overexpression promote cell malignancy in SKBR-3 cells. Cells were treated with 100 mM ethanol and transfected with STARD10 and ERBB2 plasmids or ERBB2 siRNA (10 nM)for 48 h (A) (B) MTT assay. Data are expressed as (mean ± SE) from 4 to 5 independent experiments performed in triplicates. *p < 0.04 vs. EV; *p < 0.01 vs. Sc + EV; †p < 0.02 vs. EtOH; ‡p < 0.02 vs. STARD10. (C) Migration assay. Results are shown as total wound area at 0 h and 48 h. Data are expressed as (mean ± SE) from 5 independent experiments performed in triplicates. *p < 0.05 vs. EV 48 h. (PPTX 600 kb) [file 13046_2018_1013_MOESM6_ESM.pptx]

## Slide 1
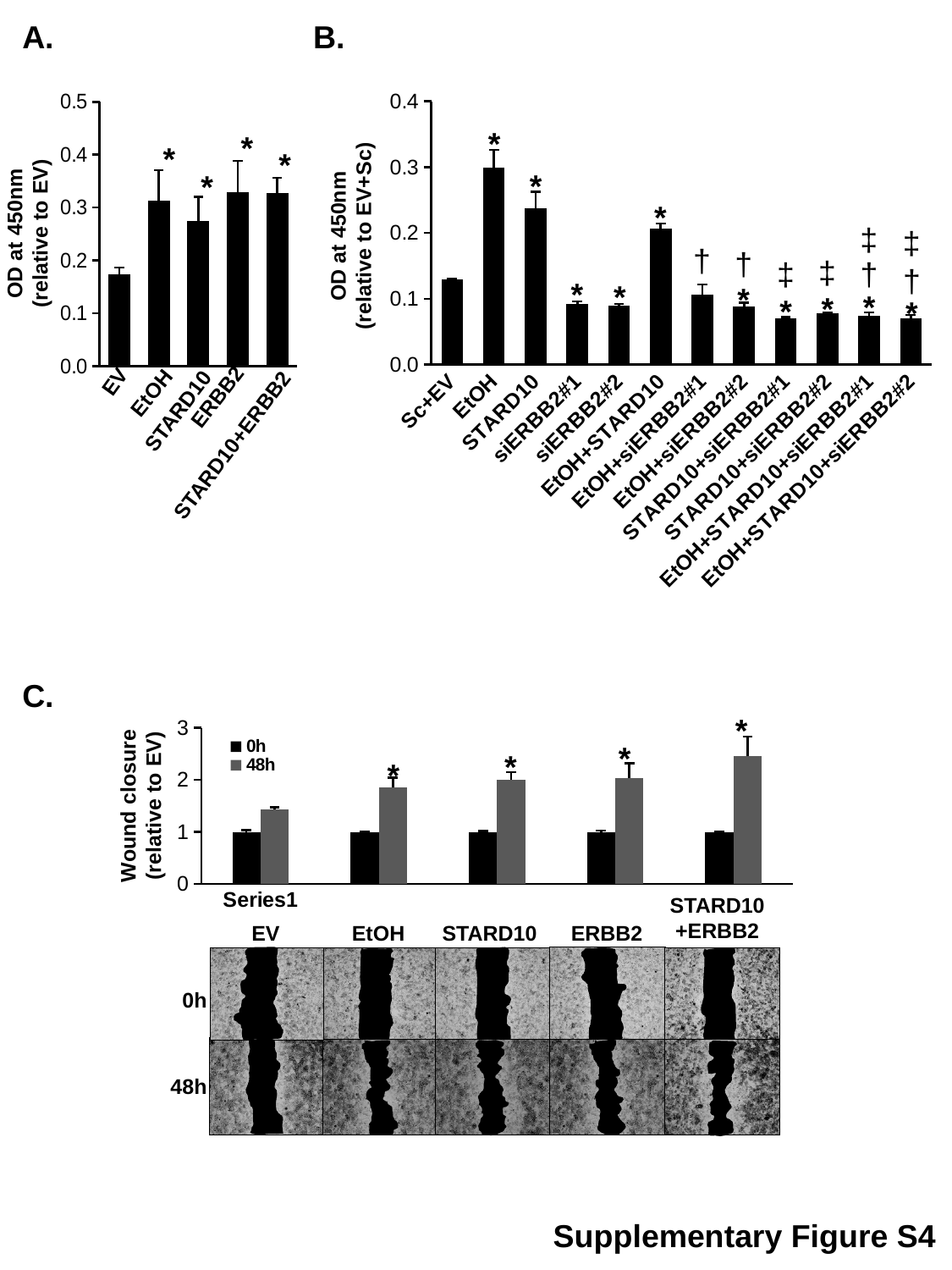

A.
B.
### Chart
| Category | |
|---|---|
| EV | 0.173125 |
| EtOH | 0.3126875 |
| STARD10 | 0.27418750000000003 |
| ERBB2 | 0.32825000000000004 |
| STARD10+ERBB2 | 0.3275 |*
*
*
*
OD at 450nm
(relative to EV)
EV
ERBB2
EtOH
STARD10
STARD10+ERBB2
*
### Chart
| Category | |
|---|---|
| Sc+EV | 0.129 |
| EtOH | 0.2985 |
| STARD10 | 0.23725000000000002 |
| siERBB2#1 | 0.0915 |
| siERBB2#2 | 0.08843749999999999 |
| EtOH+STARD10 | 0.20655 |
| EtOH+siERBB2#1 | 0.10622500000000001 |
| EtOH+siERBB2#2 | 0.08712500000000001 |
| STARD10+siERBB2#1 | 0.0694525 |
| STARD10+siERBB2#2 | 0.0768325 |
| EtOH+STARD10+siERBB2#1 | 0.0739825 |
| EtOH+STARD10+siERBB2#2 | 0.06997249999999999 |*
*
‡
OD at 450nm
(relative to EV+Sc)
‡
†
†
*
*
*
‡
†
‡
*
*
†
*
*
C.
*
### Chart
| Category | 0h | 48h |
|---|---|---|
| | 1.0 | 1.426 |
| | 1.0 | 1.86 |
| | 1.0 | 2.0033333333333334 |
| | 1.0 | 2.03 |
| | 1.0 | 2.4566666666666666 |*
*
*
Wound closure
(relative to EV)
STARD10
+ERBB2
EV
EtOH
ERBB2
STARD10
0h
48h
Supplementary Figure S4
