# Supplementary material for: Star-related lipid transfer protein 10 (STARD10): a novel key player in alcohol-induced breast cancer progression
Source: J Exp Clin Cancer Res. 2019 Jan 5;38:4. doi: 10.1186/s13046-018-1013-y (PMC6321732; doi:10.1186/s13046-018-1013-y)
Supplement: Supplementary file 7 — Figure S5. STARD10 silencing increases cell malignancy in vitro. MCF-7 and SKBR-3 cells were transfected with two different STARD10 siRNAs (10 nM) for 48 h. (A)(D) Efficiency of STARD10 silencing was determined by qRT-PCR. Data are expressed as (mean ± SE) from 3 independent experiments performed in triplicates.*p < 0.001 vs. Sc MCF-7 cells; *p < 0.001 vs. Sc SKBR-3 cells (B)(E) MTT assay showing viability of MCF-7 cells transfected with two different StarD10 silencers. Data are expressed as (mean ± SE) from 3 independent experiments performed in triplicates. *p < 0.04 vs. Sc MCF-7 cells; *p < 0.03 vs. Sc SKBR-3 cells. (C)(F) Graphs of migration assay. Data are expressed as (mean ± SE) from 4 independent experiments performed in triplicates. 48 h. Results are shown as percent of wound with compared to Sc at 0 h and 48 h. Data are expressed as (mean ± SE) from 3 independent experiments performed in triplicates. *p < 0.04 vs. Sc 0 h MCF-7 cells; †p < 0.05 vs. Sc 48 h MCF-7 cells. *p < 0.05 vs. Sc 0 h; †P < 0.05 vs. Sc 48 h SKBR-3 cells. (PPTX 112 kb) [file 13046_2018_1013_MOESM7_ESM.pptx]

## Slide 1
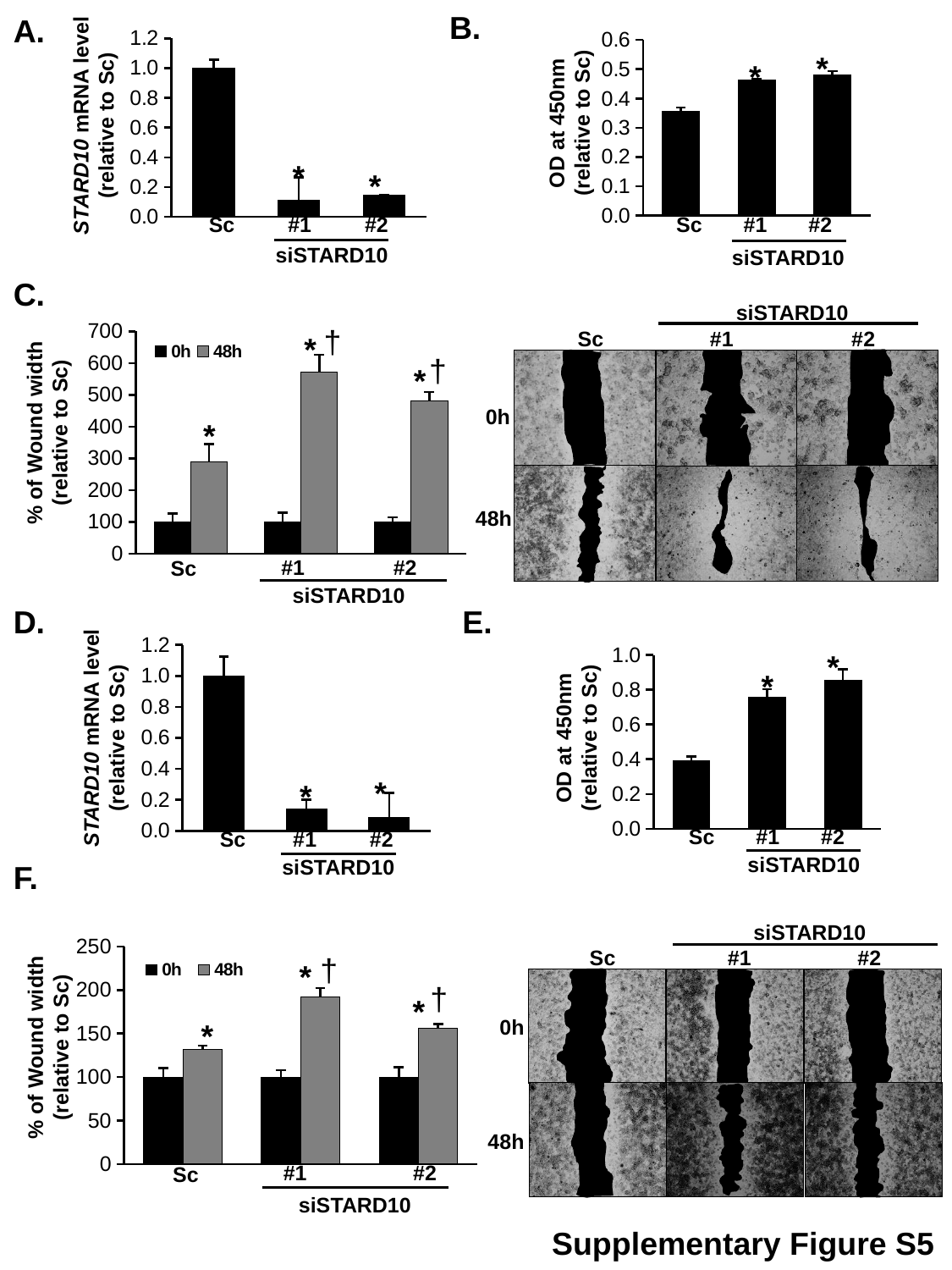

B.
A.
### Chart
| Category | |
|---|---|
| Sc | 1.0 |
| siSTARD10#1 | 0.11187813386599639 |
| siSTARD10#2 | 0.14711334215234548 |
### Chart
| Category | |
|---|---|
| Sc | 0.357 |
| siSTARD10 #1 | 0.465 |
| siSTARD10 #2 | 0.483 |*
*
STARD10 mRNA level
(relative to Sc)
OD at 450nm
(relative to Sc)
*
*
Sc #1 #2
siSTARD10
Sc #1 #2
siSTARD10
C.
siSTARD10
Sc #1 #2
0h
48h
### Chart
| Category | 0h | 48h |
|---|---|---|
| Sc | 100.0 | 288.675 |
| siStarD10#1 | 100.0 | 572.275 |
| siStarD10#2 | 100.0 | 480.99999999999994 |†
*
*
% of Wound width
(relative to Sc)
*
#1 #2
Sc
siSTARD10
D.
E.
### Chart
| Category | |
|---|---|
| Sc | 0.393 |
| siSTARD10#1 | 0.757 |
| siSTARD10#2 | 0.858 |*
*
OD at 450nm
(relative to Sc)
Sc #1 #2
siSTARD10
### Chart
| Category | |
|---|---|
| Sc | 1.0 |
| siSTARD10#1 | 0.14508798929796504 |
| siSTARD10#2 | 0.08626983458728914 |STARD10 mRNA level
(relative to Sc)
*
*
Sc #1 #2
siSTARD10
F.
siSTARD10
Sc #1 #2
0h
48h
### Chart
| Category | 0h | 48h |
|---|---|---|
| Sc | 100.0 | 132.0 |
| siSTARD10#1 | 100.0 | 192.0 |
| siSTARD10#2 | 100.0 | 156.0 |% of Wound width
(relative to Sc)
#1 #2
Sc
siSTARD10
†
*
†
*
*
Supplementary Figure S5
